# Hepatic Transcriptome Responses of Domesticated and Wild Turkey Embryos to Aflatoxin B_1_

**DOI:** 10.3390/toxins8010016

**Published:** 2016-01-06

**Authors:** Melissa S. Monson, Carol J. Cardona, Roger A. Coulombe, Kent M. Reed

**Affiliations:** 1 Department of Veterinary and Biomedical Sciences, College of Veterinary Medicine, University of Minnesota, St. Paul, MN 55108, USA; monso181@umn.edu (M.S.M.); ccardona@umn.edu (C.J.C.); 2 Department of Animal, Dairy and Veterinary Sciences, College of Agriculture, Utah State University, Logan, UT 84322, USA; roger@usu.edu

**Keywords:** aflatoxin, domesticated turkey, wild turkey, embryonic, liver, RNA-seq, differential expression

## Abstract

The mycotoxin, aflatoxin B_1_ (AFB_1_) is a hepatotoxic, immunotoxic, and mutagenic contaminant of food and animal feeds. In poultry, AFB_1_ can be maternally transferred to embryonated eggs, affecting development, viability and performance after hatch. Domesticated turkeys (*Meleagris gallopavo*) are especially sensitive to aflatoxicosis, while Eastern wild turkeys (*M. g. silvestris*) are likely more resistant. *In ovo* exposure provided a controlled AFB_1_ challenge and comparison of domesticated and wild turkeys. Gene expression responses to AFB_1_ in the embryonic hepatic transcriptome were examined using RNA-sequencing (RNA-seq). Eggs were injected with AFB_1_ (1 μg) or sham control and dissected for liver tissue after 1 day or 5 days of exposure. Libraries from domesticated turkey (*n* = 24) and wild turkey (*n* = 15) produced 89.2 Gb of sequence. Approximately 670 M reads were mapped to a turkey gene set. Differential expression analysis identified 1535 significant genes with |log_2_ fold change| ≥ 1.0 in at least one pair-wise comparison. AFB_1_ effects were dependent on exposure time and turkey type, occurred more rapidly in domesticated turkeys, and led to notable up-regulation in cell cycle regulators, NRF2-mediated response genes and coagulation factors. Further investigation of NRF2-response genes may identify targets to improve poultry resistance.

## 1. Introduction

Domesticated turkeys (*Meleagris gallopavo*) are highly susceptible to the hepatotoxic and immunotoxic effects of the mycotoxin, aflatoxin B_1_ (AFB_1_). Consumption of AFB_1_-contaminated feed by poultry can induce liver damage, immunosuppression and poor growth [1,2,3,4]. Generated in the liver, *exo*-AFB_1_-8,9- epoxide (AFBO) is the major metabolite responsible for AFB_1_ toxicity [4,5,6]. AFBO can bind to DNA and other macromolecules causing mutations, impairing transcription and translation, and initiating apoptosis [4,5,6,7]. In domesticated turkeys, hepatic cytosolic alpha-class glutathione *S*-transferase (GSTA) enzymes lack the ability to detoxify AFBO, which is likely the most important factor underlying their extreme sensitivity [4,8,9].

Unlike their domesticated relatives, Eastern wild turkeys (*M. g. silvestris*) appear relatively AFB_1_-resistant [10]. Supporting this, AFBO-conjugating activity was demonstrated for hepatic GSTA enzymes from both the Eastern and Rio Grande (*M. g. intermedia*) subspecies of wild turkey [11]. More efficient GST-mediated hepatic detoxification of AFBO could be largely responsible for greater resistance, although variation in apoptotic processes, cellular regulation, immune responses, and other pathways could also contribute. To our knowledge, no *in vivo* experiments have compared domesticated and wild turkey responses to AFB_1_ and most work has utilized *ad libitum* feeding studies that allow variation in the dose of AFB_1_ ingested by each bird. Therefore, to better characterize the differences between domesticated and wild turkeys, direct comparison of their responses to a controlled dose of AFB_1_ is needed.

This study utilized an egg injection (*in ovo*) route of AFB_1_ challenge to examine domesticated and wild turkey responses in the liver after either 1 or 5 days of exposure. Embryonic exposure ensures a precise administration of the toxin and provides a more cost effective model for toxicity assessment than in hatched poults. During egg formation, AFB_1_ can transfer from the laying hen into the egg yolk and albumen [2,12,13,14,15,16,17]. Presence of AFB_1_ in embryonated and unfertilized eggs is of concern to the poultry industry. *In ovo* AFB_1_ exposure of developing chickens (*Gallus gallus*) and turkeys results in DNA damage in the liver, morphological defects, and embryonic mortality [14,18,19,20,21,22,23,24]. Direct experimental injection of eggs or embryonic exposure through maternal feeding can also compromise cellular and humoral immune functions in the hatched progeny [19,22,25,26,27].

*In ovo* responses to AFB_1_ can be compared in embryos by analysis of differential gene expression. Comparison of domesticated and wild birds could potentially identify genes or alleles associated with decreased susceptibility to the effects of AFB_1_. Previous work in our laboratory applied RNA-sequencing (RNA-seq) to investigate AFB_1_ effects on hepatic and splenic gene expression in domesticated turkey poults after dietary exposure [28,29]. In the liver, expression changes were identified in transcripts linked to apoptosis, carcinogenesis and lipid metabolism [28]. In the spleen, both innate and adaptive immune response genes were affected [29]. RNA-seq allows for characterization of expressed sequence from the entire transcriptome, without the hybridization and inclusion bias of microarrays. This study was designed to apply RNA-seq to AFB_1_-exposed embryonic liver tissue, which will provide insight into the mechanisms of toxicity, determine the similarity between *in ovo* models and live dietary exposures, and detect differences in the responses of wild and domesticated turkeys. We hypothesized that AFB_1_ would have differential *in ovo* effects on the hepatic transcriptome of wild turkeys *versus* domesticated birds.

## 2. Results

### 2.1. Phenotypic Effects of AFB_1_

Eggs containing viable domesticated turkey (DT) and wild turkey (WT) embryos were injected with either an AFB_1_-solution (AFB) or a sham control (CNTL) and incubated for the subsequent 1 day or 5 days (four groups in each turkey type: CNTL 1 day exposure (C1), AFB 1 day exposure (A1), CNTL 5 days exposure (C5) and AFB 5 days exposure (A5)). Weight measurements were collected at the end of exposure to characterize phenotypic effects of AFB_1_ toxicity. Embryonic exposure to AFB_1_ for 5 days (A5 *versus* C5) significantly reduced mean embryo weight in WT, relative liver weight in DT, and absolute liver weights in both types of turkey (Table 1 and Table S1). No significant differences were observed in either DT or WT after 1 day of AFB_1_ exposure (A1 *versus* C1). Although the CNTL groups were not significantly different across turkey types, egg and embryo weights in WTA5 were lower than in DTA5. The expected growth and maturation of the embryos over time (C5 *versus* C1) led to significant increases in embryo and liver weights (Table S1). However, as development progressed, mean relative liver weight only increased in DT.

**Table 1:** Effect of aflatoxin B_1_ on egg, embryo, and liver weights in domesticated and wild turkeys.

Type	Exposure	Treatment ^1^	Number of Embryos	Egg Weight (g)		Embryo Weight (g)		Liver Weight (g)		Relative Liver Weight (g)	
				Mean	SD	Mean	SD	Mean	SD	Mean	SD
DT	1 Day	CNTL	7	79.56	±5.62	16.58	±0.97	0.26	±0.03	1.56	±0.12
		AFB	7	81.13	±8.49	17.67	±0.49	0.28	±0.03	1.56	±0.17
	5 Days	CNTL	7	76.6	±5.71	30.7	±1.93	0.65 ^d^	±0.13	2.12 ^f^	±0.37
		AFB	7	82.16 ^a^	±5.56	32.60 ^b^	±2.86	0.53 ^d^	±0.22	1.64 ^f^	±0.22
WT	1 Day	CNTL	4	66.77	±8.75	18.71	±1.09	0.34	±0.04	1.81	±0.20
		AFB	4	74.7	±4.51	19.08	±1.17	0.33	±0.04	1.74	±0.26
	5 Days	CNTL	3	66.3	±10.52	29.59 ^c^	±4.46	0.64 ^e^	±0.07	2.21	±0.34
		AFB	4	60.18 ^a^	±3.68	24.83 ^b,c^	±0.46	0.46 ^e^	±0.06	1.87	±0.25

Matched superscript letters ^a,b,c,d,e,f^ indicate significant differences (*p*-value < 0.05) between AFB and CNTL or DT and WT groups. All post-hoc comparisons are provided in Table S1. Domesticated turkey (DT), wild turkey (WT), control (CNTL), aflatoxin B1 (AFB), standard deviation (SD). ^1^ In ovo exposure to 30% EtOH (CNTL) or 1 µg of AFB1 (AFB).

### 2.2. RNA-seq Datasets

Total RNA isolated from DT and WT embryonic liver samples (*n* = 24 and *n* = 15, respectively) was used for construction of individual barcoded libraries. Sequencing of all libraries (*n* = 39) produced over 441 M read pairs (883 M total reads) composing 89.2 Gb of raw sequence data (Table 2). Reads (in pairs) per library averaged 22.6 M (range of 17.1–29.9 M); mean library depth was higher in WT (25.5 M) than DT (20.9 M) (Table 2 and Table S2). After filtering and trimming, corrected datasets were only slightly reduced (98.3% of raw data, average of 22.3 M reads per library, range of 16.9–29.4 M) and contained both read pairs and single reads. Characteristics of the corrected datasets in DT and WT were similar, with minimal differences in read length (95.7 bp to 95.1 bp), mean quality score (36.6 to 35.9), and mean GC content (46.9% to 46.6%) (Table 2). In both DT and WT, quality scores in corrected reads were sufficiently high across all base positions (Figure S1).

**Table 2:** RNA-seq datasets from domesticated and wild turkey embryonic liver.

Type	Number of Libraries	Read Status	Mean Read Length (bp)	Mean Quality Score	Mean GC Content (%)	Read Pairs	Single Reads	Total Sequence (Gb)
DT	24	Raw	101.0	35.8	47.2	250,429,631	NA	50.6
		Corrected	95.7	36.6	46.9	245,205,648	4,829,880	47.4
WT	15	Raw	101.0	34.7	46.9	190,989,257	NA	38.6
		Corrected	95.1	35.9	46.6	184,931,064	5,721,404	35.7

Domesticated turkey (DT), wild turkey (WT), not applicable (NA).

### 2.3. Mapping to MAKER Gene Set

Approximately 77% of the 83.1 Gb of corrected sequence mapped uniquely to the MAKER annotated turkey gene set (Table 2 and Table 3). This percentage was consistent across all treatment groups in both DT and WT (Table 3 and Table S2). In each corrected dataset, a higher percentage of paired reads mapped to the gene set than single reads (Table S2). As expected, the majority (73.3%) of mapped reads aligned to exons, rather than introns. Over half (57.8%) of these exonic reads spanned exon borders, illustrating the importance of splice junction mapping when investigating eukaryotic genomes. In total, 17,440 genes (95.5% of the gene set) were expressed in at least one group, with a mean depth of 941 reads/gene (Table 3). Gene expression across the genome was highly similar in DT and WT (Table S3). Most genes (96.8% in DT, 96.5% in WT) with known chromosomal locations were expressed; a lower 79.9% of unassigned genes were expressed in either turkey type.

**Table 3:** Read mapping to the MAKER annotated turkey gene set.

Type	Mapped Reads (% of Corrected) ^1^	Unmapped Reads (% of Corrected) ^1^	Expressed Genes (% of Total Genes)	Mean Read Depth per Gene
DT	380.6 M (76.9%)	114.6 M (23.1%)	17,293 (94.7%)	868
WT	289.4 M (77.0%)	86.1 M (22.9%)	17,211 (94.2%)	1056
Total	670.1 M (76.9%)	200.8 M (23.1%)	17,440 (95.5%)	941

Domesticated turkey (DT), wild turkey (WT). ^1^ Paired reads counted as two.

### 2.4. Sample Variation

**Figure 1:**
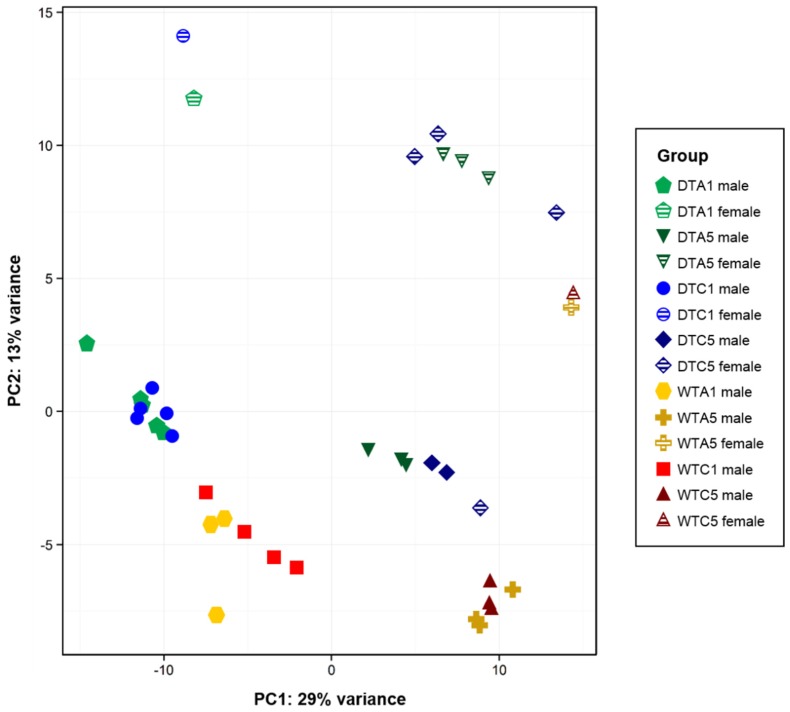
Exposure time, turkey type, and gender cause variation in embryonic transcriptomes. Principle component analysis (PCA) was performed on regularized log_2_ transformed read counts in DESeq2 [30]. Principle component 1 (PC1) and principle component 2 (PC2) explain 42% of the variation in read counts. Samples are plotted by group: DTC1 (blue circles), DTC5 (dark blue diamonds), DTA1 (green pentagons), DTA5 (dark green inverted triangles), WTC1 (red squares), WTC5 (dark red triangles), WTA1 (gold hexagons), and WTA5 (dark gold crosses). Gender for each sample is also indicated (male = solid, female = striped). Domesticated turkey (DT), wild turkey (WT), control 1 day (C1), control 5 days (C5), aflatoxin B_1_ 1 day (A1), aflatoxin B_1_ 5 days (A5).

Principle component analysis (PCA) was used to evaluate variation within and between groups based on regularized log_2_ transformed read counts (Figure 1). The principle component 1 (PC1) axis explained the greatest amount of variation (29%) and separated transcriptomic expression profiles based on exposure time (1 day and 5 days). Further clustering occurred according to turkey type, irrespective of treatment. For example, samples from WTA1 and WTC1 intermixed but were distinct from DTA1 and DTC1. All treatment groups except WTA1 and WTC1 were also divided into two clusters along principle component 2 (PC2). Further investigation clarified that this sample distribution represented differences in the liver transcriptome due to the effects of gender. In each treatment, the female embryonic samples clustered higher along PC2 than the male; only one female (DTC5L7) did not follow this pattern. Overall, this distribution illustrates the developmental differences between samples, the distinct expression profiles of DT and WT, and the effect of gender on hepatic tissue. Hierarchical clustering of the distances between samples reiterated the relationships shown by PCA (Figure S2).

### 2.5. Differential Expression Analysis

After read count normalization and differential expression (DE) analysis, nearly half (48.9%, 8929) of the genes in the MAKER gene set were identified as having significant DE (*q*-value ≤ 0.05) in at least one pair-wise comparison between groups. However, only a small portion of these genes (17.2%, 1535) had expression changes greater than |log_2_ fold change (log_2_FC)| ≥ 1.0, illustrating the stability of embryonic expression in the liver (Table S4; Figures S3 and S4). This stability of expression profiles could be due to conservation of developmental processes across turkey types (DT *versus* WT), to similarities in the late stages of embryonic development (1 day *versus* 5 days), and to the contained environment of the egg minimizing variation and limiting AFB_1_ effects to direct toxicity (AFB *versus* CNTL). Significant genes with greatest DE (|log_2_FC| ≥ 1.0) also had the highest significance in each pair-wise comparison. Table S4 provides the full list of significant DE genes with |log_2_FC| ≥ 1.0 in at least one comparison. Of these genes, 814 (53.0%) were unique to a single pair-wise comparison, although three genes (absent in melanoma 1-like protein (*AIM1L*), coiled-coil domain-containing protein 135 (*CCDC135*), and cytochrome P450 2H2 (*CYP2H2*)) were identified in seven of twelve comparisons (Table S4). Analysis of each pair-wise comparison elucidates DE attributed to development, AFB_1_ exposure and genetic differences between DT and WT.

### 2.6. Developmental Effects

Comparison between the control groups at 1 day and 5 days can provide important information on gene expression during embryonic development. As development progressed in DT (DTC5 *versus* DTC1), 19.7% (3593) of genes in the MAKER gene set were significantly DE (*q*-value ≤ 0.05; log_2_FC from −2.8 to 3.7). Expression of the majority of these genes (82.2%) had small changes in expression and only 640 genes had |log_2_FC| ≥ 1.0 (Table S4; Figure S5). Significant genes with large expression changes were primarily (71.4%) up-regulated during development, with the greatest increase observed in megakaryoblastic leukemia/myocardin-like protein 1 (*MKL1*) and decrease in cytochrome P450 1A5 (*CYP1A5*) (Table S4). To best characterize affected pathways, functional analysis in Ingenuity Pathway Analysis (IPA) utilized DE data from all significant genes in the comparison. In DT, IPA assigned the highest significance to multiple signaling pathways, including “non-small cell lung cancer signaling” and “pancreatic adenocarcinoma signaling” (Table S5; Figure S6A). Genes in these pathways (including those with non-significant DE) were predominately down-regulated during development (Figure S6A) and likely reflect decreases in expression of genes involved in cellular functions and development. Exposure to AFB_1_ over time (DTA5 *versus* DTA1) reduced the significance of these pathway associations, showing that introduction of the toxin can affect normal developmental processes.

In WTC5 *versus* WTC1, 2313 genes (12.7% of the gene set) had significant DE (log_2_FC from −2.9 to 3.0), of which 316 (13.7%) had |log_2_FC| ≥ 1.0 (Table S4; Figure S5). More than half (61.7%) of the significant genes with large expression changes were up-regulated, with the greatest DE observed in protein phosphatase 1 regulatory subunit 3B-like (*PPR3B*; up-regulated) and ATP-binding cassette sub-family G member 8-like (*ABCG8*; down-regulated). Associations to “protein ubiquitination pathway” and “RAN signaling” had the highest significance in IPA (Table S5; Figure S6B). Similar to changes in DT, the majority of DE genes in these pathways (44.0% and 63.2%, respectively) were down-regulated in WT (Figure S6B). Introduction of AFB_1_ decreased or eliminated the significance of these pathway associations (WTA5 *versus* WTA1). Multiple genes (173) were significant and had |log_2_FC| ≥ 1.0 in both DT and WT comparisons of control groups, illustrating conserved changes during embryo development (Table S4; Figure S5). Examples of highly DE genes observed in both DT and WT include *PPR3B*, neuroligin-1 (*NLGN1*), *ABCG8*, and *CYP1A5*.

Direct comparisons between DT and WT were also made in the CNTL groups at each exposure time. In WTC1 *versus* DTC1, 66 genes had significant DE with |log_2_FC|≥ 1.0 (1593 total significant DE; log_2_FC from −1.7 to 3.2) (Table S4). Poly(U)-specific endoribonuclease (*ENDOU*) was the most up-regulated gene in WT, while disintegrin and metalloproteinase domain-containing protein 9 (*ADAM9*) was the most down-regulated. Fewer genes (1044) had significant DE (log_2_FC from −3.6 to 2.6) at the later developmental time point (WTC5 *versus* DTC5); however, 94 had |log_2_FC| ≥ 1.0. Glutamate receptor-interacting protein 1 (*GRIP1*; up-regulated) and an unannotated gene (down-regulated) had the greatest log_2_FC. The most significant pathway associations in WTC1 *versus* DTC1 were observed for “pancreatic adenocarcinoma signaling” and “coagulation system” (Table S5). Other signaling pathways had highly significant associations at the later time point, illustrating that differences in gene expression profiles in domesticated and wild turkeys changed as development progressed.

### 2.7. Effects of 1 Day of Exposure to AFB_1_

**Figure 2:**
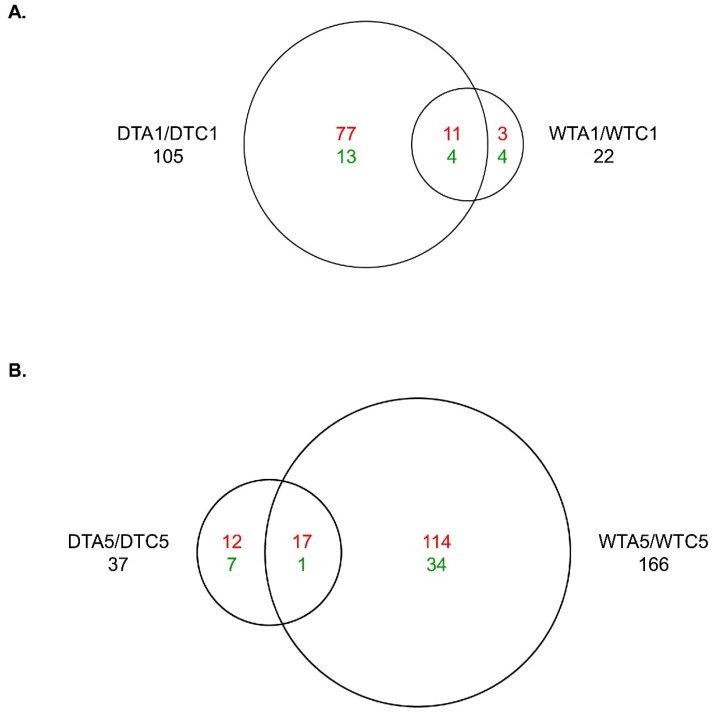
Treatment-related expression changes from aflatoxin B_1_ (AFB_1_) occurs predominately in DTA1 and WTA5. (**A**) A1 *versus* C1 in DT and WT. (**B**) A5 *versus* C5 in DT and WT. Each diagram shows the number of shared and unique genes with significant differential expression (DE) (*q*-value ≤ 0.05) and |log_2_FC| ≥ 1.0. Direction of DE (red, above = up-regulated, and green, below = down-regulated) is shown. Domesticated turkey (DT), wild turkey (WT), control 1 day (C1), control 5 days (C5), aflatoxin B_1_ (AFB_1_), aflatoxin B_1_ 1 day (A1), aflatoxin B_1_ 5 days (A5), log_2_ fold change (log_2_FC).

DE analysis comparing the AFB_1_-exposed group in DT (DTA1) *versus* the control (DTC1) identified 2144 genes with significant DE (*q*-value ≤ 0.05; log_2_FC from −1.8 to 4.6). In total, 105 (4.9%) had |log_2_FC| ≥ 1.0 (Table S4; Figure 2A); these were predominately (83.8%) up-regulated by AFB_1_. The greatest increases in expression were observed for cyclin dependent kinase inhibitor CIP1 (*CIP1/CDKN1A/p21*), ectodysokasin A2 receptor (*EDA2R/TNFRSF27*), S-adenosylmethionine synthase isoform type-1 (*MAT1A*), and E3 ubiquitin-protein ligase MDM2 (*MDM2*) (Table S4). Genes showing the greatest down-regulation after 1 day of exposure in DT were RNA binding protein fox-1 homolog 1 (*RBFOX1*) and hydrocephalus-inducing protein homolog (*HYDIN*).

**Table 4:** Significant DE of genes regulated by nuclear factor erythroid 2-related factor 2-like (NRF2) in AFB *versus* control (CNTL) groups.

Gene ID	Gene	DTA1/DTC1		DTA5/DTC5		WTA1/WTC1		WTA5/WTC5	
		Log_2_FC	*q*-value	Log_2_FC	*q*-value	Log_2_FC	*q*-value	Log_2_FC	*q*-value
T_ALT_S_008999	*AKR1A1*	0.34	3.18 × 10^−2^	0.07	7.76 × 10^−1^	0.25	8.07 × 10^−1^	0.69	5.58 × 10^−5^
T_ALT_S_014672	*AKR7A2*	0.47	1.15 × 10^−2^	0.14	4.91 × 10^−1^	0.34	5.55 × 10^−1^	0.18	3.37 × 10^−1^
T_ALT_S_008024	*AOX1*	0.28	3.65 × 10^−2^	0.00	9.96 × 10^−1^	−0.02	9.95 × 10^−1^	−0.04	8.86 × 10^−1^
T_ALT_S_003995	*CAT*	−0.18	3.15 × 10^−1^	−0.44	3.49 × 10^−4^	−0.18	8.54 × 10^−1^	−0.30	1.58 × 10^−1^
T_ALT_S_010665	*DNAJA3*	0.40	1.15 × 10^−3^	−0.03	8.79 × 10^−1^	0.07	9.78 × 10^−1^	0.21	1.66 × 10^−1^
T_ALT_S_009842	*DNAJB11*	−0.32	7.44 × 10^−3^	−0.08	NA	−0.39	2.36 × 10^−1^	0.31	1.15 × 10^−1^
T_ALT_S_008078	*DNAJC10*	−0.39	1.01 × 10^−3^	−0.02	9.65 × 10^−1^	−0.05	9.91 × 10^−1^	0.18	4.09 × 10^−1^
T_ALT_S_014426	*DNAJC11*	0.26	4.81 × 10^−2^	−0.06	7.63 × 10^−1^	−0.20	8.47 × 10^−1^	0.20	3.88 × 10^−1^
T_ALT_S_007708	*DNAJC13*	0.03	8.56 × 10^−1^	−0.40	6.56 × 10^−3^	0.07	9.80 × 10^−1^	0.08	7.48 × 10^−1^
T_ALT_S_014061	*DNAJC18*	−0.02	8.95 × 10^−1^	−0.02	9.44 × 10^−1^	−0.24	5.93 × 10^−1^	−0.42	1.81 × 10^−2^
T_ALT_S_002734	*EPHX1*	0.63	5.74 × 10^−4^	−0.06	8.95 × 10^−1^	0.03	9.93 × 10^−1^	0.57	2.36 × 10^−3^
T_ALT_S_003948	*FTH1*	0.10	6.77 × 10^−1^	0.38	4.52 × 10^−2^	−0.10	9.41 × 10^−1^	0.12	7.29 × 10^−1^
T_ALT_S_003347	*GSTA3* ^1^	1.60	1.47 × 10^−10^	0.74	1.79 × 10^−2^	0.48	3.63 × 10^−1^	0.52	2.34 × 10^−1^
T_ALT_S_003348	*GSTA3* ^1^	1.52	2.87 × 10^−9^	0.85	4.31 × 10^−3^	0.39	5.52 × 10^−1^	0.23	6.61 × 10^−1^
T_ALT_S_003346	*GSTA3* ^1^	1.11	9.74 × 10^−5^	0.40	2.66 × 10^−1^	0.36	5.83 × 10^−1^	0.80	4.76 × 10^−2^
T_ALT_S_003345	*GSTA4*	0.82	8.65 × 10^−7^	0.47	2.82 × 10^−2^	0.35	6.56 × 10^−1^	0.42	4.09 × 10^−2^
T_ALT_S_001023	*GSTK1*	0.07	6.99 × 10^−1^	−0.31	2.39 × 10^−2^	−0.26	5.83 × 10^−1^	−0.11	6.34 × 10^−1^
T_ALT_S_009475	*GSTO1*	0.13	5.87 × 10^−1^	0.06	8.65 × 10^−1^	−0.28	3.27 × 10^−1^	1.07	4.63 × 10^−3^
T_ALT_S_011992	*HERPUD1*	−0.25	3.76 × 10^−1^	0.69	3.75 × 10^−4^	−0.20	9.21 × 10^−1^	−0.26	3.11 × 10^−1^
T_ALT_S_000662	*HMOX1*	0.29	1.91 × 10^−1^	−0.14	7.31 × 10^−1^	0.32	3.84 × 10^−1^	0.85	1.48 × 10^−9^
T_ALT_S_005601	*MGST2*	0.84	6.76 × 10^−7^	0.14	6.23 × 10^−1^	0.41	2.18 × 10^−1^	0.37	8.19 × 10^−2^
T_ALT_S_008700	*MGST3*	0.56	9.79 × 10^−5^	0.10	7.83 × 10^−1^	0.33	2.55 × 10^−1^	0.73	4.64 × 10^−4^
T_ALT_S_012166	*NQO1*	0.63	2.64 × 10^−6^	0.03	8.87 × 10^−1^	0.57	1.81 × 10^−3^	0.02	9.39 × 10^−1^
T_ALT_S_004785	*NQO2*	0.24	2.42 × 10^−1^	0.63	1.94 × 10^−4^	0.00	1.00	0.73	6.78 × 10^−6^
T_ALT_S_009000	*PRDX1*	0.49	2.13 × 10^−3^	0.10	6.39 × 10^−1^	0.10	9.69 × 10^−1^	0.66	1.03 × 10^−4^
T_ALT_S_016734	*STIP1*	−0.03	8.93 × 10^−1^	−0.19	2.64 × 10^−1^	−0.14	8.98 × 10^−1^	0.36	9.48 × 10^−3^
T_ALT_S_007072	*TXN*	0.09	5.73 × 10^−1^	−0.62	2.26 × 10^−3^	−0.05	9.80 × 10^−1^	−0.07	6.80 × 10^−1^
T_ALT_S_000674	*TXNRD1* ^1^	0.61	1.32 × 10^−2^	0.15	7.63 × 10^−1^	0.54	2.55 × 10^−1^	0.45	2.82 × 10^−1^
T_ALT_S_011677	*TXNRD1* ^1^	−0.30	2.07 × 10^−3^	0.00	9.99 × 10^−1^	0.00	9.99 × 10^−1^	−0.16	3.06 × 10^−1^
T_ALT_S_007907	*UGT1A1*	0.62	2.35 × 10^−5^	0.06	8.19 × 10^−1^	0.15	9.05 × 10^−1^	0.34	8.16 × 10^−3^
T_ALT_S_009396	*MRP2*	0.16	3.07 × 10^−1^	−0.32	3.68 × 10^−2^	0.24	4.82 × 10^−1^	0.24	2.59 × 10^−1^
T_ALT_S_006174	*SOD3*	0.33	3.12 × 10^−1^	0.17	7.08 × 10^−1^	0.07	9.92 × 10^−1^	0.58	2.25 × 10^−2^

Genes down-stream of nuclear factor erythroid 2-related factor 2-like (NRF2) were identified using Ingenuity Pathway Analysis (IPA). Log_2_ fold change (Log_2_FC) and FDR-adjusted *p*-values (*q*-values) were determined in DESeq2 [30]. Non-significant comparisons (*q*-value > 0.05) are shown in grey. Differential expression (DE), domesticated turkey (DT), wild turkey (WT), control (CNTL), control 1 day (C1), control 5 days (C5), aflatoxin B_1_ (AFB), aflatoxin B_1_ 1 day (A1), aflatoxin B_1_ 5 days (A5), no statistics due to low read counts (NA). ^1^ Multiple genes in the MAKER gene set had significant DE and annotated to the same reference.

**Figure 3:**
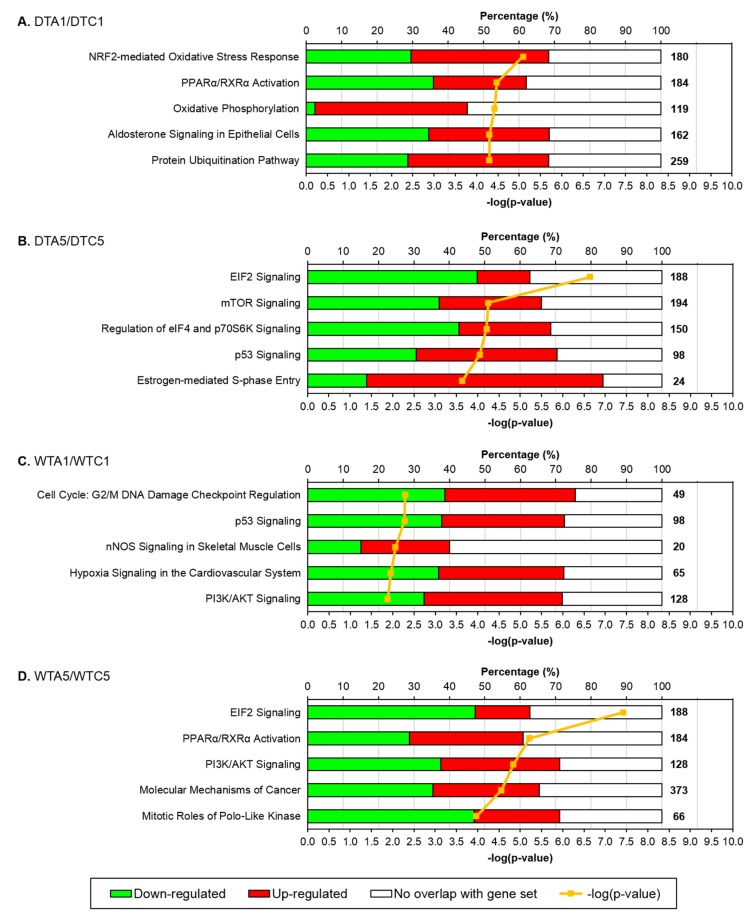
Differential expression in highly significant canonical pathways in comparisons of AFB *versus* CNTL groups. (**A**) DTA1/DTC1. (**B**) DTA5/DTC5. (**C**) WTA1/WTC1. (**D**) WTA5/WTC5. Ingenuity Pathway Analysis (IPA) was used to identify significant effects on canonical pathways (−log(*p*-value) > 1.3). Percentage of genes down-regulated (green), up-regulated (red), not represented in MAKER annotated gene set (white) are shown for the 5 most significant pathways (−log(*p*-values) in yellow) in each pair-wise comparison; both significant and non-significant DE is included. Total numbers of genes in each pathway are in bold. Domesticated turkey (DT), wild turkey (WT), control (CNTL), control 1 day (C1), control 5 days (C5), aflatoxin B_1_ (AFB), aflatoxin B_1_ 1 day (A1), aflatoxin B_1_ 5 days (A5).

**Figure 4:**
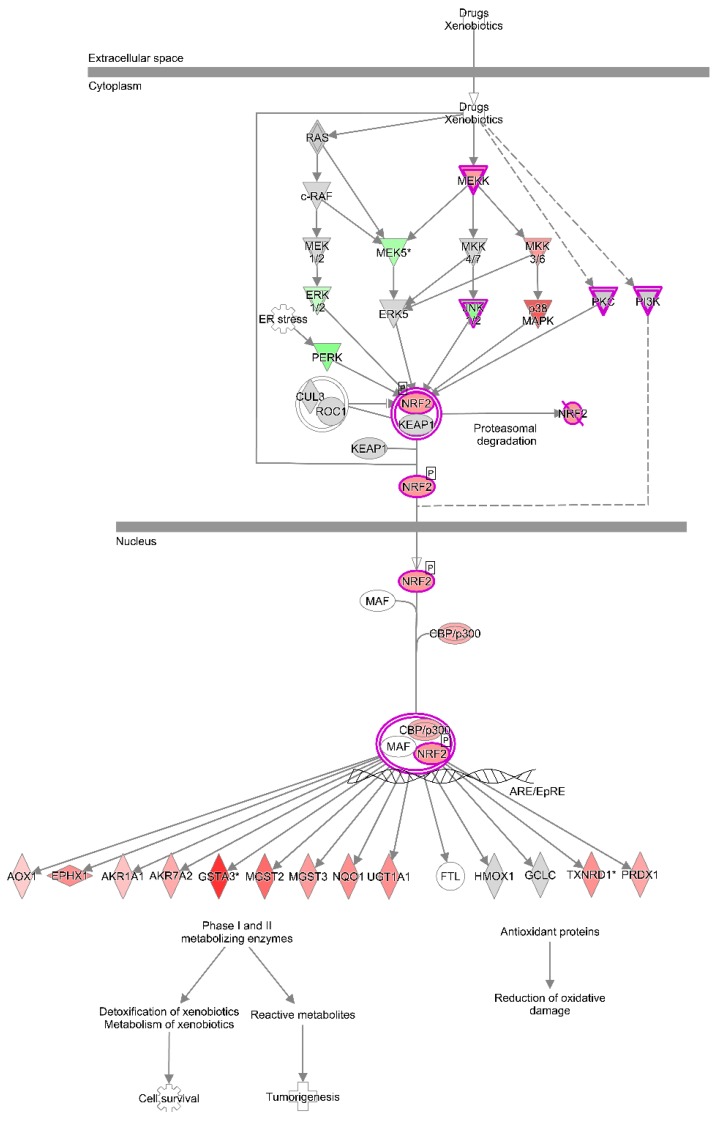
Up-regulation of NRF2-mediated responses in DTA1 *versus* DTC1. Ingenuity Pathway Analysis (IPA) identified highly significant association of this pathway (*p*-value of 8.22 × 10^−6^). Genes were up-regulated (red), down-regulated (green), not significant (grey), or not represented in the MAKER gene set (white). Purple outlines represent groups of molecules and indicate gene annotations that occur more than once in the gene set. Nuclear factor erythroid 2-related factor 2-like (NRF2), domesticated turkey (DT), control 1 day (C1), aflatoxin B_1_ 1 day (A1).

The highest IPA associations in the DTA1/DTC1 comparison were made to the “NRF2-mediated oxidative stress response”, “PPARα/RARα activation” and “oxidative phosphorylation” pathways (Table S5; Figure 3A). AFB_1_ exposure increased expression of many genes in these pathways (Figure 3A). For example, 38.9% of genes in the NRF2-mediated oxidative stress response pathway were up-regulated (both significant and non-significant DE) in DTA1. Although significant, nuclear factor erythroid 2-related factor 2-like (*NFE2L2*/NRF2) itself was only moderately up-regulated (Table S6; Figure 4). Expression of many downstream targets of this transcription factor increased in DT after 1 day exposure to AFB_1_ (Table 4 and Table S6; Figure 4). Highest significant up-regulation was observed in alpha-class glutathione *S*-transferase 3 (*GSTA3*), alpha-class glutathione *S*-transferase 4 (*GSTA4*), and microsomal glutathione *S*-transferase 2 (*MGST2*) (Table 4). It should be noted that three genes in the MAKER gene set are annotated to *GSTA3*; each representing a fragment of the known sequence [31]. This likely results from misassembly in this repetitive genomic region. Increased expression was also seen in other phase I and II metabolic enzymes and anti-oxidant proteins, including microsomal glutathione *S*-transferase 3 (*MGST3*), aflatoxin B1 aldehyde reductase 2 (*AKR7A2*/*AFAR*), epoxide hydrolase 1 (*EPHX1*), NAD(P)H dehydrogenase quinone 1 (*NQO1*), thioredoxin reductase 1 cytoplasmic (*TXNRD1*) and UDP-glucuronosyltransferase 1-1 (*UGT1A1*).

In contrast to the DT, 1 day exposure to AFB_1_ in WT (WTA1 *versus* WTC1) had limited effects on gene expression; only 201 genes (1.1%) in the MAKER gene set had significant DE (log_2_FC from −1.7 to 2.8). Among these, 22 (10.9%) had |log_2_FC| ≥ 1.0 and 8 were down-regulated (Table S4; Figure 2A). Similar to DT, the most up-regulated gene in WT was *MDM2* (Table S4). The most significant IPA associations in the WTA1/WTC1 comparison were to “G2/M DNA damage checkpoint regulation” and “p53 signaling”, illustrating misregulation of the cell cycle after AFB_1_ exposure in WT (Table S5; Figure 3C). Despite the vast difference in the numbers of significant genes, 15 genes were in common between the DT and WT and shared even the direction of expression changes following exposure (Figure 2A). For instance, *MDM2*, E3 ubiquitin-protein ligase MYCBP2 (*MYCBP2*), *RBFOX1*, and thioredoxin-like protein AAED1 (*AAED1*) were affected in both types of turkey (Table S4). Therefore, even with the lower AFB_1_ sensitivity of WT, some expression changes in the initial response to AFB_1_ were conserved with DT.

### 2.8. Effect of 5 Days of Exposure to AFB_1_

After 5 days of exposure to AFB_1_ in DT (DTA5 *versus* DTC5), 1036 genes had significant DE (*q*-value ≤ 0.05; log_2_FC from −1.8 to 3.9), but only 37 had |log_2_FC| ≥ 1.0 (Table S4, Figure 2B). AFB_1_ increased expression of 78.4% (29) of the genes with |log_2_FC| ≥ 1.0 and many of these genes were also up-regulated by 1 day exposure (e.g., *AAED1*, *CIP1*, *MAT1A*, *MDM2* and *EDA2R*). As in DTA1, *CIP1* had the greatest increase in expression after 5 days exposure (DTA5), although the log_2_FC was slightly lower (Table S4). “EIF2 signaling”, “mTOR signaling”, and other signaling pathways were the most significantly associated IPA pathways after 5 days of exposure in DT (Table S5; Figure 3B).

The number of significant genes (1904; log_2_FC from −1.9 to 4.6) in WTA5 verses WTC5 was greater than that observed in the DTA5/DTC5 comparison. Nearly 4.5 times as many genes in WT (166) had |log_2_FC| ≥ 1.0 (Table S4, Figure 2B). The largest log_2_FC values were observed for *EDA2R*, *MAT1A*, *CIP1* and cytochrome P450 2C45 (*CYP2C45*) (Table S4). Consistent with the other comparisons involving the AFB group, 78.9% of genes with significant DE and |log_2_FC| ≥ 1.0 were up-regulated (Table S4, Figure 2B). Pathway associations with the highest significance in WT after 5 days of exposure were “EIF2 signaling” and “PPARα/RARα activation” (Table S5; Figure 3D), both of which were highly significant in other AFB to CNTL group comparisons. Eighteen significant and highly DE genes were identified in both WTA5/WTC5 and DTA5/DTC5, including the three with the greatest DE (*EDA2R*, *MAT1A* and *CIP1*) (Table S4, Figure 2B).

### 2.9. Gender Effects

Gender also warranted further examination in DE analysis since it contributed to the clustering of samples observed in PCA (Figure 1). Since gender was unequally represented in most comparisons, only DTA5 *versus* DTC5 was evaluated using gender as a second factor in DESeq2. When incorporating gender, the number of genes with significant DE in DTA5/DTC5 slightly increased (1102 genes; log_2_FC from −1.8 to 3.9). However, the number of significant DE genes (39) with |log_2_FC| ≥ 1.0 was consistent with the original analysis (Tables S4 and S7). Most (36) of the significant genes with |log_2_FC| ≥ 1.0 were identified irrespective of gender as a factor. Among the 980 genes with significant DE in both analyses, gender only determined whether three genes met the log_2_FC threshold. Furthermore, only one gene (annexin A4 (*ANXA4*)) had significant DE and |log_2_FC| ≥ 1.0 in the two-factor analysis, but did not have significance in the original comparison (*q*-value of 1.86 × 10^−1^).

Thirty genes (76.9%) were found in the two-factor analysis to be significantly up-regulated (significant DE and |log_2_FC| ≥ 1.0) by AFB_1_ exposure (Table S7). Three genes, (*CIP1*, *EDA2R*, and *MAT1A*) had the largest positive log_2_FC values, while tubulointerstitial nephritis antigen-like (*TINAG*) was the most down-regulated (Table S7). This is in complete agreement with the original comparison of DTA5/DTC5. Incorporating gender into the analysis also had limited effect on the canonical pathway associations identified by IPA (Table S5). Overall, the identity and functions of significant genes were highly consistent in both analyses, demonstrating that gender had minimal effects on AFB_1_-induced expression changes despite its role in the transcriptome as a whole. Therefore, due to the unbalanced distribution across groups and the congruence of both DTA5/DTC5 comparisons, gender was not included as a secondary factor in the overall DE analysis.

### 2.10. Comparison of AFB_1_ Effects

Differences in transcriptome responses to AFB_1_ exposure were examined in direct comparisons between WT and DT. In WTA1 *versus* DTA1, 716 genes had significant DE (log_2_FC from −1.9 to 2.8; 45 with |log_2_FC| ≥ 1.0), while 2050 were significant (log_2_FC from −3.3 to 2.9; 248 with |log_2_FC| ≥ 1.0) in WTA5 *versus* DTA5 (Table S4). Some of the detected differences between WT and DT occurred irrespective of treatment (CNTL or AFB). For example, similar to the CNTL comparisons, *ENDOU* was one of the most highly up-regulated genes in the WTA1/DTA1 and WTA5/DTA5 comparisons. A large increase in *GRIP1* was also observed in both AFB and CNTL comparisons, but only at the later time point in AFB-exposed birds (WTA5 *versus* DTA5). However, pathway analysis identified greater effects on the “oxidative phosphorylation” and “mitochondrial dysfunction” pathways in the WTA5/DTA5 comparison than WTC5/DTC5 (Figure S7). In WTA1 *versus* DTA1, “DNA double-strand break repair by non-homologous end joining” had a significant pathway association not observed in other comparisons between turkey types.

**Figure 5:**
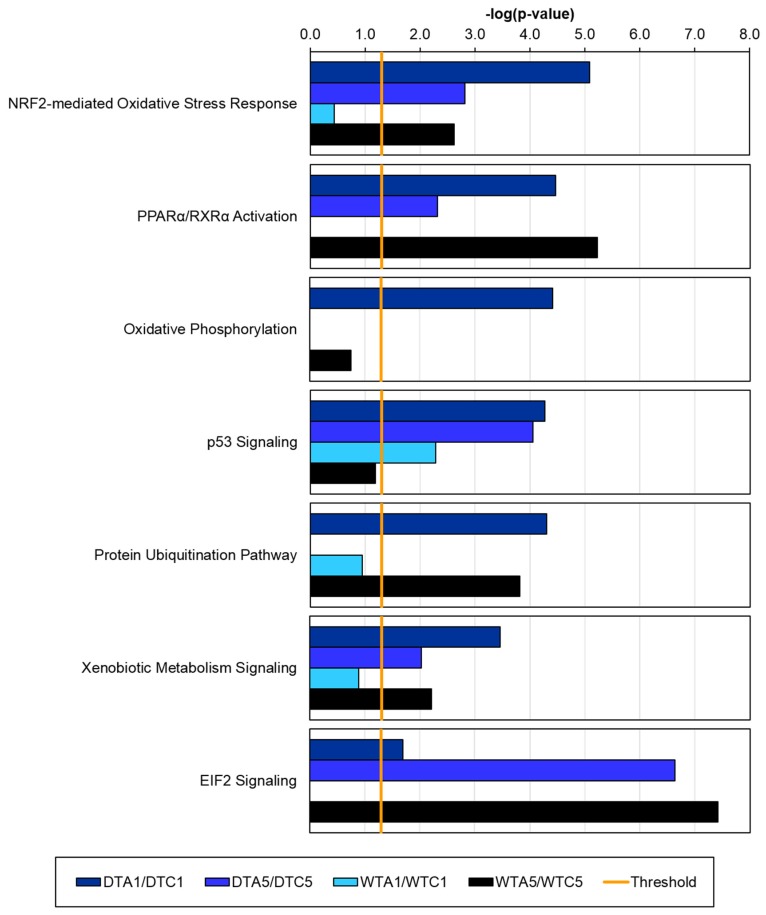
Significance of pathway associations vary in AFB *versus* CNTL comparisons. In each pair-wise comparison, Ingenuity Pathway Analysis (IPA) assigned *p*-values to canonical pathways based on differential expression (DE). Bar plot provides 6 example pathways with variable significance between the DTA1/DTC1 (dark blue), DTA5/DTC5 (bright blue), WTA1/WTC1 (light blue), and WTA5/WTC5 (black) comparisons. Pathway associations must have a -log(*p*-value) > 1.3 (threshold, vertical yellow line) to be considered significant. Domesticated turkey (DT), wild turkey (WT), control (CNTL), control 1 day (C1), control 5 days (C5), aflatoxin B_1_ (AFB), aflatoxin B_1_ 1 day (A1), aflatoxin B_1_ 5 days (A5).

Differences in the pathways affected by AFB_1_ in DT and WT were also highlighted by comparative pathway analysis in IPA (Figure 5). For example, “EIF2 signaling” was significant after 5 days of exposure to AFB_1_ in both DT and WT, due to similar levels of down-regulation of genes in this pathway (47.9% and 47.3%, respectively) (Figure 3 and Figure 5). The “PPARα/RARα activation” and “protein ubiquitination” pathways occurred in the comparisons with greatest AFB_1_ effects (DTA1/DTC1 and WTA5/WTC5). Significant associations were made to the “NRF2-mediated oxidative stress response” pathway in all AFB *versus* CNTL comparisons except WTA1/WTC1 (Figure 5). In these comparisons (DTA1/DTC1, DTA5/DTC5, and WTA5/WTC5), there were similar levels of up-regulated (37.8%–38.9%) and down-regulated (28.3%–30.0%) genes in the NRF2-response pathway. Interestingly, the gene encoding NRF2 (*NFE2L2*) was up-regulated in both DT time points and in WTA1, but not in the WTA5 group (Table S6). The significance of DE in downstream targets of NRF2 was even more variable (Table 4 and Table S6). After 5 days of exposure, *GSTA3*, homocysteine-responsive endoplasmic reticulum-resident ubiquitin-like domain member 1 protein (*HERPUD1*), and dehydrogenase quinone 2 (*NQO2*) were most up-regulated in DT, while *GSTA3*, heme oxygenase 1-like (*HMOX1*), *MGST3*, and *NQO2* increased most in WT. In WTA1 *versus* WTC1, *NQO1* was the only downstream gene with significant DE (up-regulated).

Toxicological function analysis in IPA also identified significant associations to hepatotoxic outcomes that reflect the known phenotypic effects of AFB_1_ exposure. In all pair-wise comparisons of AFB *versus* CNTL, “liver hyperplasia” had highest significance (Figure S8). The strength of the associations also mirrored the overall effects of AFB_1_ on expression, with highest *p*-values in DTA1/DTC1 and WTA5/WTC5 and lowest in the WTA1/WTC1 comparison. Outcomes such as “hepatocellular carcinoma” and “liver steatosis” also had significant associations in most comparisons. These potential hepatotoxic outcomes fit with expected tissue-wide effects, while canonical pathways and individual expression changes illustrate differential responses to AFB_1_ exposure in WT and DT.

## 3. Discussion

This study utilized an *in ovo* exposure model to characterize gene expression responses to AFB_1_ in the hepatic transcriptome of domesticated and wild turkeys. Responsible for phase II detoxification, the liver is also the primary site of AFB_1_ activation into AFBO and its resulting toxicity [4,5,6]. Consequently, *in ovo* AFB_1_ exposure of chickens and domesticated turkeys induces dose-related DNA damage in the embryonic liver [24] and can reduce embryo viability [14,18,19,20,21,22,23,24]. Although no embryo mortality was seen in this study, *in ovo* AFB_1_ exposure had phenotypic effects and altered expression in the hepatic transcriptome.

### 3.1. Responses to AFB_1_

Dietary AFB_1_ exposure in poultry leads to lipid accumulation in the liver, which causes hepatomegaly and increased liver weight relative to body weight in both chickens and domesticated turkeys [1,2,3,32,33,34,35,36,37,38,39]. In the present study, embryonic exposure to AFB_1_ decreased the relative weight in domesticated turkey embryos. That liver mass was not increased in AFB_1_-exposed embryos is not surprising given the short duration of exposure. A short-term decline in relative liver weight has been reported in broilers fed dietary aflatoxins; as the study progressed, both lipids and relative liver weights increased [35]. In this study, embryo weights also significantly decreased after *in ovo* exposure in wild turkeys, but not domesticated turkeys. Both AFB_1_ injection and maternal feeding have been shown to reduce embryo weight in chickens [14,20,21]. In addition to the observed phenotypic changes, hepatic transcriptome analysis identified significant effects on genes involved in cell cycle regulation and NRF2-mediated responses.

Genes linked to the cell cycle and apoptosis were consistently among the most significant and up-regulated genes in AFB_1_-exposed groups. For example, expression of *MDM2*, *CIP1* and *MAT1A* were significantly increased in domesticated and wild turkey embryos in AFB *versus* CNTL comparisons (*CIP1* and *MAT1A* were not significant in WTA1/WTC1). AFB_1_-induced expression changes in these genes have been identified in multiple species, suggesting that AFB_1_ effects on the cell cycle are conserved. Up-regulation of *MDM2* after AFB_1_ exposure has been observed in human hepatocellular carcinoma (HCC) cells [40], rats [41], swine [42], and in our previous study of turkey poults [28]. Increased hepatic expression of *CIP1* in response to AFB_1_ has been seen in human primary hepatocytes or HCC cell lines [40,43,44], mice [45], rats [41], swine [42], and turkeys [28]. Interestingly, *MAT1A* expression has been shown to decrease in swine [42] and human HCC cells [40] after AFB_1_ exposure.

Inhibition of apoptotic pathways or misregulation of the cell cycle by these genes could facilitate the development of hepatic lesions in AFB_1_-exposed poultry, including vaoculation of hepatocytes, necrotic loci, focal hemorrhages, biliary hyperplasia, fibrosis and nodular tissue regeneration [1,2,33,34,36,46,47,48,49]. MDM2 is an E3 ubiquitin-protein ligase known to mark the tumor suppressor p53 for degradation by the proteasome [50,51]. However, overexpression of *MDM2* can downregulate p53 in cells that should undergo apoptosis, causing aberrant cell cycle progression. *CIP1* encodes a p53-activated inhibitor of cyclin-dependent kinase activity that regulates cell cycle progression. Overexpression of CIP1 could lead to cell cycle arrest and apoptosis [42], contrary to the proliferative effects of MDM2-mediated p53 loss. Increased expression of these regulatory genes during short-term embryonic exposure in the turkey suggests that the apoptotic and hyper-proliferative phenotypes seen in the liver begin to develop in the early stages of aflatoxicosis.

Expression of *S*-adenosylmethionine synthase could affect liver health and sulfur metabolism. In mammals, *MAT1A* and S-adenosylmethionine synthase isoform type-2 (*MAT2A*) encode interchangeable subunits that metabolize methionine for use in DNA methylation [52,53,54]. MAT enzymes can also regulate hepatocyte proliferation and apoptosis [52,53] and affect glutathione production [54]. Mammalian MAT1A is the normal hepatic form of the enzyme, while MAT2A is expressed extrahepatically, as well as in fetal liver and HCC [52,53,54]. In a recent study from our laboratory, *MAT2A* was also up-regulated in AFB_1_-fed domesticated turkey poults [28]. Conversely, up-regulation of *MAT1A* after AFB_1_ exposure in this study could occur as part oxidative stress responses; increased MAT expression could feed additional S-adenosylmethionine into glutathione synthesis. However, three genes in the turkey MAKER gene set used in this study annotate as MAT synthases (two to *MAT1A* and one to *MAT2A*). Further clarification of their identity, normal expression patterns, and the functional consequences of AFB_1_-induced differential expression is needed to fully understand the role of these enzymes in poultry.

Based on expression changes in the embryonic liver, cellular responses to oxidative stress and xenobiotics were also initiated by AFB_1_ exposure. *NFE2L2* encodes the NRF2 leucine zipper transcription factor central to many of these responses; this gene was up-regulated after 1 day of exposure to AFB_1_ in both domesticated and wild turkey embryos. After activation by phosphorylation, NRF2 binds anti-oxidant response elements (AREs) in nuclear DNA and activates transcription of metabolic/detoxifying enzymes, anti-oxidants and anti-apoptotic factors [55,56,57,58]. Mammalian NRF2 can be stabilized in its active form by interacting with CIP1 [56,59]. In this study, significant up-regulation of *NFE2L2* and *CIP1* was observed in the same comparisons of embryonic exposure to AFB_1_, suggesting a similar interaction could be possible in poultry.

*GSTs* are NRF2-response genes with a critical role in domesticated turkey susceptibility to AFB_1_ toxicity [4,8,9]. In embryonic turkey liver, *GSTA3* and *GSTA4* were significantly up-regulated in all AFB *versus* CNTL comparisons except WTA1/WTC1. In mice, efficient glutathione conjugation by murine GSTA3 causes high resistance to aflatoxicosis [6,60,61]. Hepatic GST enzymes in domesticated turkey are essentially unable to detoxify AFBO *in vitro*, whereas wild turkey GST enzymes retain anti-AFBO activity [4,8,9,11]. However, as the greatest up-regulation in *GSTA* genes in this study was observed in DTA1/DTC1, the lack of AFBO detoxification by hepatic GSTs does not appear to reflect an inability to respond to AFB_1_ exposure. Rather post-translational modifications may be responsible for the inactivity of GSTAs against AFBO in domesticated turkeys [8]. It is important to note that while hepatic GST enzymes are produced in turkey embryos [62]; their AFB_1_-conjugating activity has not been examined. Furthermore, since AFB_1_ exposure can lead to oxidative stress and lipid peroxidation [63,64,65], up-regulation of *GSTA* genes may reflect antioxidant functions of glutathione instead of AFBO detoxification.

Many other genes regulated by NRF2 also encode phase I and II enzymes that could be involved in the metabolism of AFB_1_ in turkey liver. In WTA5/WTC5, omega-class glutathione *S*-transferase 1 (*GSTO1*) was the most up-regulated NRF2-response gene. Up-regulation of *AFAR* was unique to DTA1/DTC1, while *EPHX1* was significant in both DTA1 and WTA5. Mammalian homologs for both of these enzymes can metabolize AFB_1_ or one of its metabolites into AFB_1_-dihydrodiol [6,66,67,68]. AFAR activity has been demonstrated in turkey liver [69,70]. The role of EPHX1 is uncertain in both birds and mammals. NRF2 also regulates genes that encode antioxidant enzymes, including *NQO1* and *NQO2* [71]; expression of these genes was time point dependent, with significant DE in both turkey types for *NQO1* after 1 day of exposure and for *NQO2* after 5 days. Differences in the response of *GSTs* and other NRF2-response genes between domesticated and wild turkeys could play a role in determining their resistance to AFB_1_ toxicity.

### 3.2. Time of Response in WT and DT

One of the overall differences in AFB_1_ treatment between wild and domesticated turkey embryos is the time-dependency of response. More significant DE was observed after 5 days of exposure in wild turkey embryos, while expression in domesticated turkeys changed quickly (1 day exposure). After only 4 hours of exposure to AFB_1_, liver DNA damage has been seen in chicken and domesticated turkey embryos; however, less damage was observed in the longer 4 days of exposure [24]. The rapid effects of AFB_1_ on domesticated turkey embryonic expression in this study could be explained by effective production of AFBO or insufficient hepatic GST detoxification. The slower expression response in wild turkey embryos could be due to a delay in toxicity from either lower levels of AFBO production or better detoxification. We are currently investigating P450-mediated activation of AFB_1_ in wild *versus* domesticated turkey liver to better understand AFB_1_ metabolism in both types of turkey. Other response pathways could also vary in the wild birds. Further investigation of phenotypic or biochemical measures of toxicity, such as hepatic lesions, DNA damage or AFB_1_-adduct production, alongside expression effects will be needed to clarify the differences in wild turkeys.

It should be noted that the effects of longer exposure (5 days) were more detrimental to the growth of wild turkey than domesticated turkey embryos. Feeding AFB_1_ to both domesticated and wild turkey poults has been previously shown to decrease growth [1,3,10]. Embryonic exposure to AFB_1_ can impair developmental processes identified in the CNTL groups (DTC5/DTC1 and WTC5/WTC1), as shown by decreased pathway associations in AFB-treated groups (DTA5/DTA1 and WTA5/WTA1). Furthermore, pathway analysis identified differences in oxidative phosphorylation and mitochondrial dysfunction in the direct comparison of WTA5 *versus* DTA5. Differences in the oxidative capabilities of the mitochondria in muscle have been shown to effect growth in chickens [72,73] and AFB_1_ is known to adversely affect hepatic mitochondrial enzymes and oxidative phosphorylation [74,75]. Therefore, if AFB_1_ effects on mitochondrial functions extend throughout the embryo, these changes may explain the decreased growth of the wild turkeys during development.

Expression changes during development may also contribute to the greater effects of AFB_1_ seen after 1 day of exposure in domesticated turkeys. For example, cytochrome P450 1A5 (*CYP1A5*) was significantly down-regulated in developmental comparisons (except WTA5 *versus* WTA1 due to an outlier). Cytochrome P450 1A5 (and 3A37) can efficiently activate AFB_1_ into AFBO *in vitro* [76,77,78,79]. The activity of hepatic P450 enzymes and therefore AFB_1_ sensitivity is inversely related to age in turkey poults [1,34,69,76]. An active protein from the CYP1A family has been identified in embryonic liver [62], suggesting that hepatotoxicity in turkey embryos may also be driven by P450 1A5. In this study, decreased expression of *CYP1A5* over time may indicate that domesticated turkeys are more susceptible to toxicity earlier in development.

### 3.3. In ovo Exposure as Model of Aflatoxicosis

Another objective of this study was to evaluate whether *in ovo* injection of AFB_1_ could be used to model expression changes induced by dietary exposure in poults. Our laboratory has previously characterized transcriptome responses in the liver and spleen of young domesticated turkeys fed AFB_1_ [28,29]. AFB_1_ had a predominately up-regulatory effect on gene expression in both the embryonic and poult AFB_1_ exposures. Significant expression changes were identified in both hepatic RNA-seq experiments for genes or transcripts involved in apoptosis, signaling, and cell cycle regulation [28]. Direct numerical comparisons of expression level between previous studies on poults and the present study cannot be made since different study designs and techniques were utilized (*de novo* assembly of predicted transcripts *versus* mapping to an annotated gene set). However, *MDM2*, *CIP1*, *EDA2R*, growth differentiation factor 15-like (*GDF15*), keratin, type II cytoskeletal cochleal-like (*K2CO*) and serine/threonine-protein kinase RIO3 (*RIOK3*) were significantly differentially expressed after AFB_1_ exposure in both the previous experiment [28] and in DTA1 and WTA5 in this study. Less genes shared significant expression changes in DTA5 (*MDM2*, *CIP1*, *EDA2R*, and *GCF15*) and WTA1 (*MDM2*, *GDF15*, and *RIOK3*), likely due to the reduced effects of AFB_1_ on these groups.

Embryonic exposure in the spleen did not recapitulate the effects of dietary AFB_1_ on the splenic transcriptome previously observed in poults [29]. Matched embryonic spleens were collected and the RNA sequenced alongside the hepatic samples described in this study. However, only 8 significant genes with |log_2_FC| ≥ 1.0 were identified in the spleen and only for the WTA1/WTC1 comparison [80]. This same group comparison had minimal expression changes in the liver. Birds rely principally on maternally transferred antibodies until immune system development is complete after hatch [81,82]. Thus, the lack of response in the spleen is likely attributable to the immature status of the organ. Therefore, effects of *in ovo* AFB_1_ exposure on immune gene expression would be better investigated after hatch.

## 4. Experimental Section

### 4.1. Embryos and Toxin Preparation

Embryonic AFB_1_ exposures were performed according to protocols approved by the Institutional Biosafety Committee (IBC) at the University of Minnesota (IBC Protocol Number: 1302-30324H). Willmar Poultry Co. (Willmar, MN, USA) generously provided fertilized commercial turkey eggs at day 14 of incubation. Eastern wild turkey eggs (*Meleagris gallopavo silvestris*) at day 0 of incubation were purchased from Stromberg’s Chicks and Game Birds (Pine River, MN, USA). Throughout the experiment, eggs were incubated at 37.0 ± 0.5 °C with approximately 40% humidity and rotation every 2 h. AFB_1_-solution was made by directly suspending AFB_1_ (Sigma-Aldrich, St. Louis, MO, USA) in 100% EtOH and diluting to a final concentration of 5 μg/mL in 30% EtOH.

### 4.2. In ovo AFB_1_ Exposure

Eggs were candled prior to the start of the exposure period to verify viability. Fewer embryos were used for wild turkey (WT) (*N* = 15) than domesticated turkey (DT) (*N* = 28) in this experiment as a result of lower fertilization rates in the wild birds. Viable eggs were randomly divided into 4 treatment groups (7 domesticated eggs/group and 4 wild eggs/group): control 1 day exposure (C1), AFB_1_ 1 day exposure (A1), control 5 days exposure (C5), and AFB_1_ 5 days exposure (A5). Due to a shorter incubation period in DT than WT, control (CNTL) and AFB_1_ (AFB) treatments were performed on developmentally equivalent days. Thus on day 17 (DT) and day 19 (WT), 0.2 mL of 30% EtOH was sterilely injected into the air sac of each egg in the C1 and C5 groups, while eggs in the A1 and A5 groups received an injection of 0.2 mL of AFB_1_-solution (1 μg of AFB_1_/egg). After 1 day of exposure, DT (day 18) and WT (day 20) in the A1 and C1 groups embryos were sacrificed. Egg, embryo, and liver weights were measured and liver tissue was collected directly into RNAlater (Ambion, Inc., Austin, TX, USA). Tissue samples were perfused overnight at 4 °C and stored at −20 °C to preserve RNA. Only 3 WT eggs could be processed for group A1 due to the death of one embryo prior to the start of the exposure period. After 5 days of exposure (day 22 and day 24 for DT and WT, respectively), eggs in the A5 and C5 groups were processed as described for the 1 day exposure groups. Three-way ANOVA and simultaneous tests for general linear hypotheses (multiple comparisons tests) were performed on weight measurements in R using the multcomp package [83,84].

### 4.3. RNA Isolation and Sequencing

Total RNA was isolated from each liver sample by TRIzol extraction (Ambion, Inc., Austin, TX, USA), DNase-treated (Turbo DNA-freeTM Kit, Ambion, Inc., Austin, TX, USA), and stored at −80 °C. Spectrophotometry (Nanodrop 1000, Nanodrop Technologies, Wilmington, DE, USA) was used for an initial assessment of RNA concentration and quality. RNA samples were submitted for QC, library preparation and sequencing at the University of Minnesota Genomics Center (UMGC). Each sample was fluorometrically quantified by RiboGreen Assay (Invitrogen Corp., Carlsbad, CA, USA) and RNA integrity was confirmed on the 2100 Bioanalyzer (Agilent Technologies, Santa Clara, CA, USA) or the LabChip GX (Caliper Life Sciences, Inc., Hopkinton, MA, USA). Samples from 6 DT embryos/group (*n* = 24) with the highest RNA quality were selected for sequencing. All WT liver RNA samples were utilized (*n* = 15) due to the smaller sample sizes. Each sequenced sample possessed an RNA Integrity Number (RIN) between 5.9 and 7.4 or an RNA Quality Score (RQS) between 5.9 and 7.9. All RNA samples had clear separation of the 18S and 28S peaks on the electropherograms. Indexed libraries were constructed with 1 μg of total RNA/sample with the TruSeq RNA Sample Preparation Kit version 2 (Illumina, Inc., San Diego, CA, USA), and size selected for approximately 200 bp inserts. Libraries were multiplexed (see Table S2 for lane and flow cell for each sample) and sequenced on the HiSeq 2000 (Illumina, Inc., San Diego, CA, USA) to produce 100-bp paired-end reads.

### 4.4. Read Filtering, Trimming, and Dataset QC Analysis

De-multiplexed RNA-seq datasets have been accessioned as part of SRA project ID: SRP067990 (see Table S2 for individual dataset IDs). Raw reads were filtered and trimmed using CLC Genomics Workbench 7.5 (CLCGWB, CLC Bio, Cambridge, MA, USA) according to the following protocol. TruSeq adapter sequences were removed and 4 bp were trimmed from the 3’ end of each read to reduce end-quality dips. Any reads less than 40 bp were discarded. Reads were rechecked for adapter sequence and trimmed for low sequence quality (limit 0.05 error probability and maximum of 2 ambiguities) and passed through a final length filter (discarded reads <40 bp). Quality of each dataset before and after processing was measured with CLCGWB and FastQC [85].

### 4.5. Read Mapping to MAKER Gene Set

Corrected reads were mapped onto a gene set [86] created on the turkey genome (UMD 5.0) using the MAKER pipeline [87]. Summaries of the MAKER gene set are provided in Table 5 and Table S2. Genes in the MAKER gene set were identified using BLAST alignment to the NCBI RefSeq and Uniprot SwissProt databases [88]. Mapping to the annotated genome was performed in CLCGWB with the standard parameters (length fraction and similarity fraction of 80%, mismatch cost of 2, insertion cost of 3 and deletion cost of 3). Paired read distances were calculated by CLCGWB and each read in a pair was counted separately (pair = 2) to allow direct comparison to single reads and inclusion of broken pairs (reads separated during mapping). Both exonic and intronic matches were counted in order to include reads that map to unannotated exons or genes with incorrect exon borders. However, only reads that mapped uniquely to a single gene were included in counts; reads that mapped to multiple locations were ignored, as their actual source could not be determined.

**Table 5:** MAKER gene set for the turkey genome (UMD 5.0).

Statistic	Number of Genes per Chromosome ^1^	% Annotated Genes per Chromosome ^1^	Number of Exons per Gene	Genomic Region (Including Introns)	Exons Only
Min	2	87.40%	1	6	6
Mean	553	93.90%	10	24,805	2298
Max	2228	100.00%	141	1,524,779	55,896
N50	N/A	N/A	N/A	57,428	3654
Total	18,265	93.70%	180,541	453,055,619	41,981,535

^1^ Gene distribution and BLAST annotation across the genome are shown in Table S3.

### 4.6. Differential Expression and Functional Analysis

Read counts were used for pair-wise comparisons between treatment groups in the R package DESeq2 following the standard workflow [30]. Read counts were first fit to a model based on a negative binomial distribution and normalized by size-scaling for differences in library sequencing depth. Empirical Bayes shrinkage estimates of dispersion and log_2_ fold change (log_2_FC) were employed by DESeq2 to prevent over-dispersion, equalize the dynamic range of read counts, handle variable sample sizes, and make log_2_FC reproducible. Differential expression (DE) of genes between groups was then evaluated with Wald inference tests using these shrinkage estimates and normalized read counts. Genes must have a *q*-value (FDR adjusted *p*-value based on the Benjamin-Hochberg procedure) ≤ 0.05 to be considered statistically significant DE in each pair-wise comparison. Significant transcripts were also filtered for a minimum |log_2_FC| ≥ 1.0. To evaluate the effects of gender, DTA5 *versus* DTC5 was re-analyzed in DESeq2 using the same parameters, except a second factor was incorporated (design = ~ gender + treatment). Gender of embryos was determined by PCR using sex-specific primer sets [89,90].

Principle component analysis (PCA), MA plots and Venn diagrams were created in R to visualize the expression data and the results of significance testing as previously described in Monson *et al.* [28,29]. Hierarchical clustering of CNTL or AFB samples (based on Euclidean sample distances) was performed in R using regularized log_2_ transformed reads counts [30]. For all significant DE genes in each pair-wise comparison (including those with |log_2_FC| < 1.0), gene pathways and toxicological functions were investigated using Ingenuity Pathway Analysis (IPA) (Ingenuity Systems, Redwood City, CA, USA).

## 5. Conclusions

Pathways and genes with significant differential expression identified in this study provide the first direct comparison of the responses of domesticated and wild turkeys to AFB_1_. Transcriptome responses to AFB_1_ occurred more rapidly in domesticated than in wild embryos, perhaps as a result of differences in AFB_1_ metabolism. The most significant effects observed in the AFB_1_-exposed groups highlighted conserved responses of cell cycle regulators, such as *MDM2* and *CIP1*. Although most evident in the early response of domesticated birds, NRF2-mediated responses to AFB_1_ were present in both domesticated and wild turkey embryos with varied effects in down-stream detoxifying and anti-oxidant enzymes. Further investigation of these NRF2-response genes may help identify underlying differences in AFB_1_ sensitivity between domesticated and wild turkeys. Overall, *in ovo* exposure to AFB_1_ successfully induced expression changes in the liver, recapitulated hepatic expression effects observed in poults fed AFB_1_, and identified similarities and differences in transcriptome response between domesticated and wild turkeys.

## Supplementary Materials

The following are available online at www.mdpi.com/2072-6651/8/1/16/s1.
