# Supplementary material for: Hepatic Transcriptome Responses of Domesticated and Wild Turkey Embryos to Aflatoxin B1
Source: Toxins (Basel). 2016 Jan 6;8(1):16. doi: 10.3390/toxins8010016 (PMC4728538; doi:10.3390/toxins8010016)
Supplement: Supplementary file 1 [file toxins-08-00016-s001.zip › toxins-110706-suppl..pdf]

Supplementary Materials: Hepatic Transcriptome Responses of Domesticated and Wild Turkey Embryos to Aflatoxin B_1

Melissa S. Monson, Carol J. Cardona, Roger A. Coulombe and Kent M. Reed

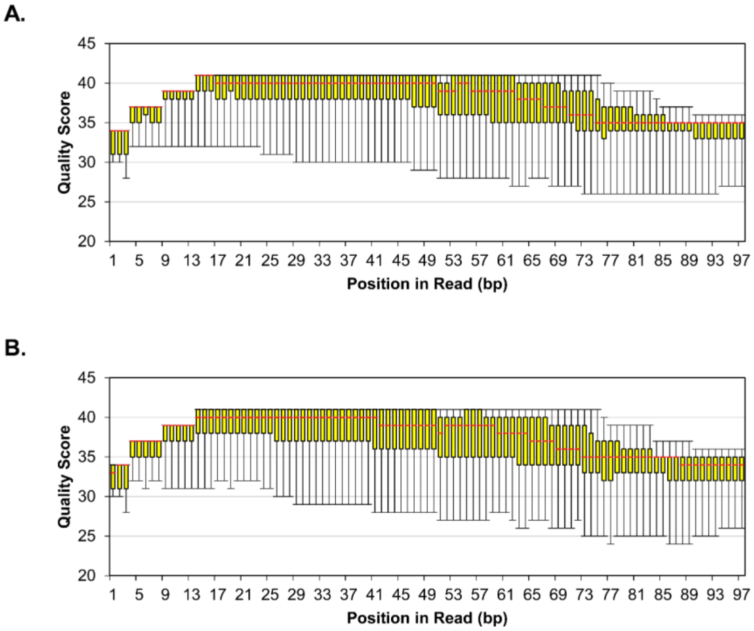

Figure S1. Quality scores at each base position for corrected liver RNA-seq datasets. (**A**) Domesticated turkey (DT); (**B**) Wild turkey (WT). Boxplots were based on quality score distributions generated in CLGWB. The median at each position is shown in red.

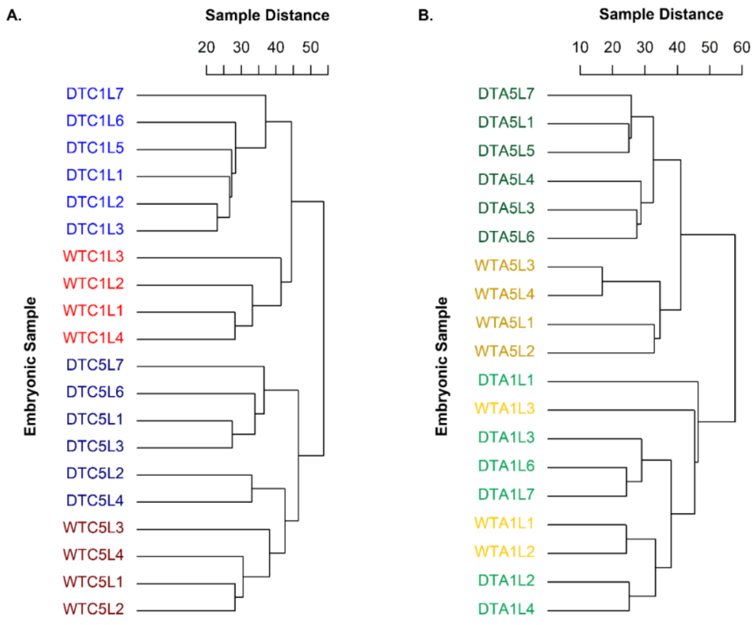

Figure S2. Hierarchical clustering of CNTL or AFB samples follows exposure time and turkey time. (**A**) Sample distances in CNTL; (**B**) Sample distances in AFB. Dendrogram was generated in R using regularized $\log_2$ transformed read counts from DESeq2 [30]. Domesticated turkey (DT, green), wild turkey (WT, yellow), aflatoxin B_1 (AFB), aflatoxin B_1 1 day (A1, lighter), aflatoxin B_1 5 days (A5, darker), liver sample (L).

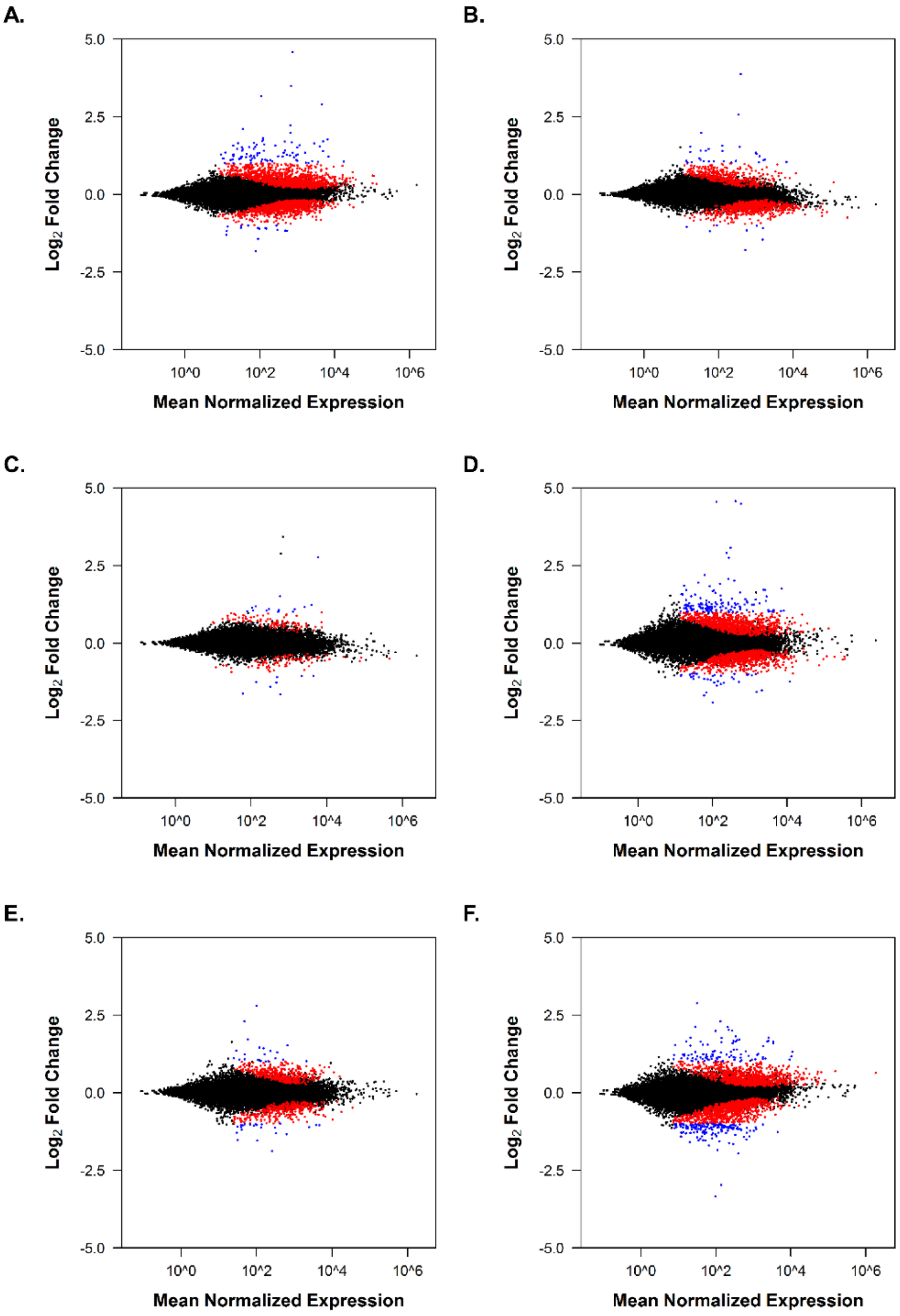

Figure S3. Significant DE from AFB exposure in DT and WT. (**A**) DTA1/DTC1; (**B**) DTA5/DTC5; (**C**) WTA1/WTC1; (**D**) WTA5/WTC5; (**E**) WTA1/DTA1; (**F**) WTA5/DTA5. MA plots were created in R using $\log_2$ fold change ($\log_2$FC) and mean normalized read counts from DESeq2 [30]. Genes with significant differential expression (DE) (*q*-values ≤ 0.05) are red; significant genes with $|\log_2FC| \geq 1.0$ are blue. Domesticated turkey (DT), wild turkey (WT), control (CNTL), control 1 day (C1), control 5 days (C5), aflatoxin B_1 (AFB), aflatoxin B_1 1 day (A1), aflatoxin B_1 5 days (A5).

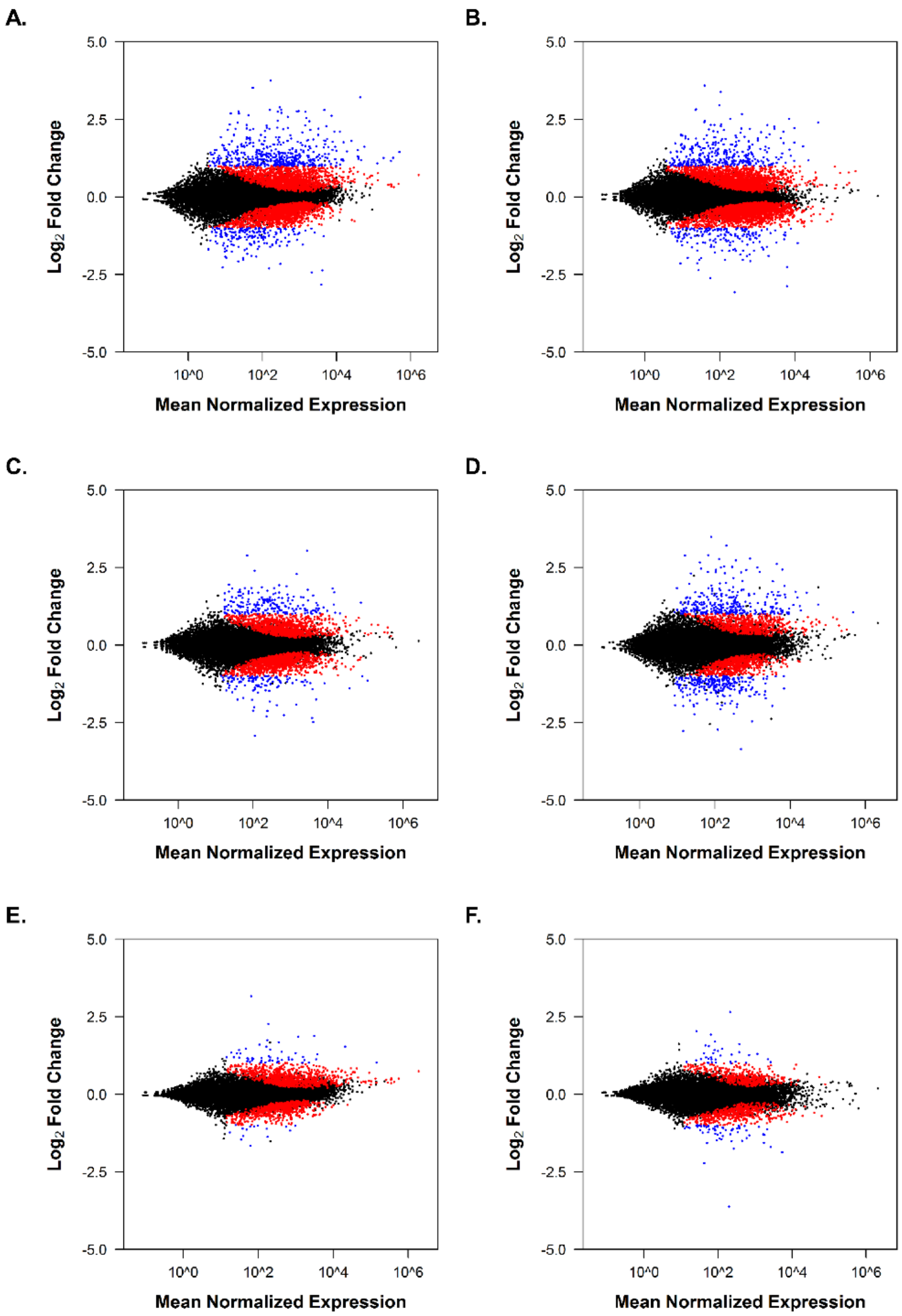

Figure S4. Significant DE during development in DT and WT. (**A**) DTC5/DTC1; (**B**) DTA5/DTA1; (**C**) WTC5/WTC1; (**D**) WTA5/WTA1; (**E**) WTC1/DTC1; (**F**) WTC5/DTC5. MA plots were created in R using $\log_2$ fold change ($\log_2$FC) and mean normalized read counts from DESeq2 [30]. Genes with significant differential expression (DE) (*q*-values ≤ 0.05) are red; significant genes with $|\log_2 FC| \geq 1.0$ are blue. Domesticated turkey (DT), wild turkey (WT), control (CNTL), control 1 day (C1), control 5 days (C5), aflatoxin B_1 (AFB), aflatoxin B_1 1 day (A1), aflatoxin B_1 5 days (A5).

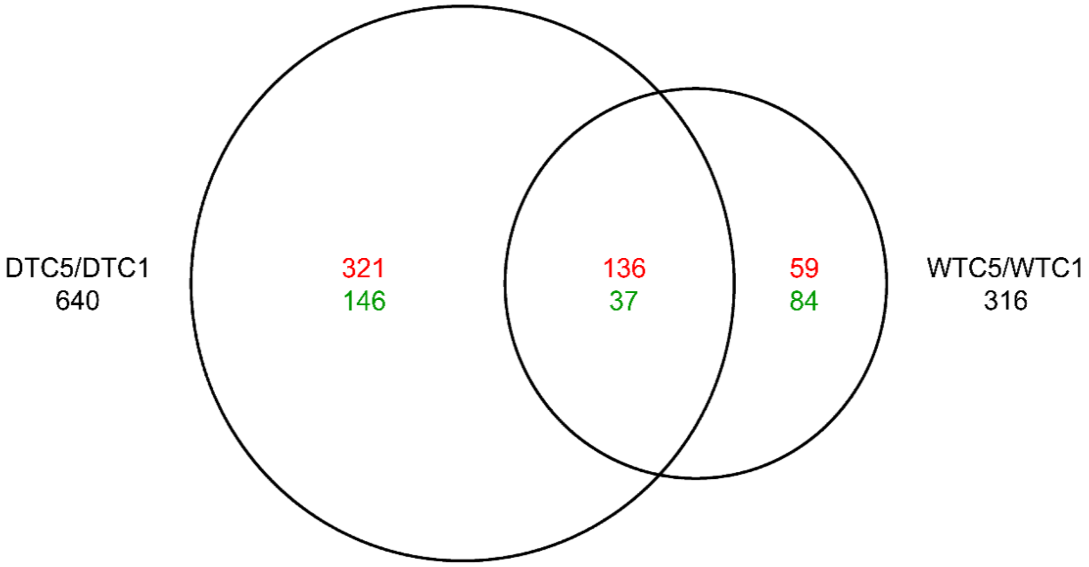

Figure S5. Overlap between significant DE in DT and WT during development. The number of shared and unique genes with significant differential expression (DE) (q-value ≤ 0.05) and $|\log_2\mathrm{FC}| \geq 1.0$ is indicated for C5 versus C1. Direction of DE is shown (red = up-regulated, green = down-regulated). Domesticated turkey (DT), wild turkey (WT), control 1 day (C1), control 5 days (C5), $\log_2$ fold change ($\log_2$FC).

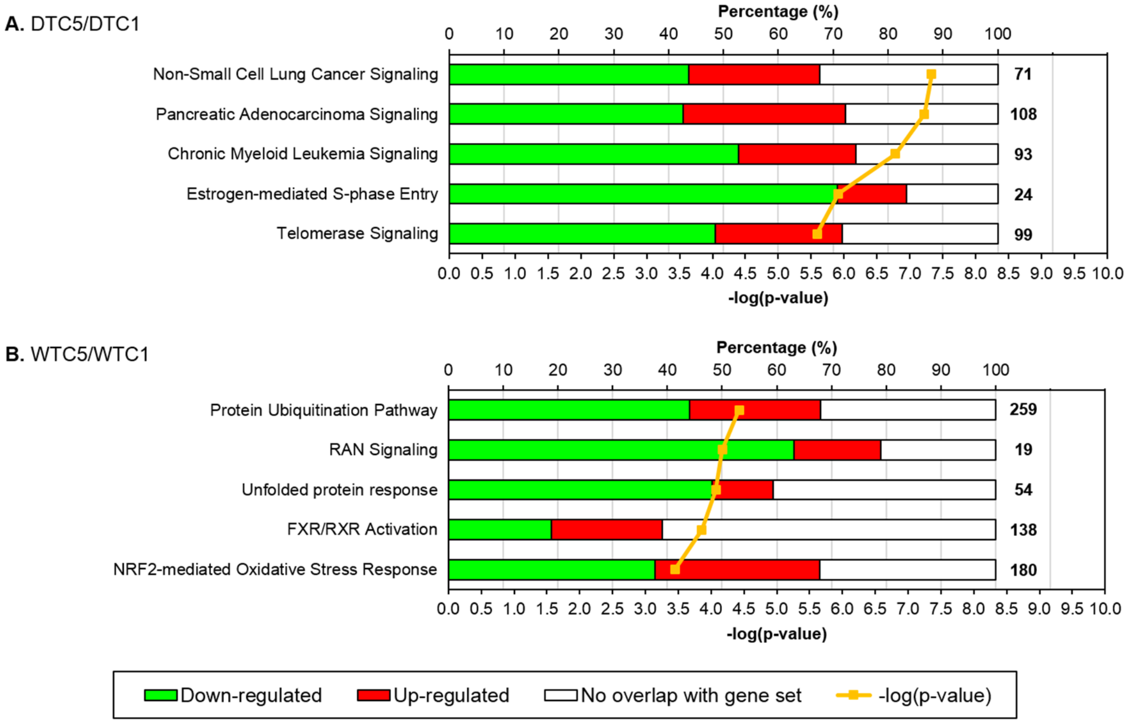

Figure S6. Expression across highly significant pathways in CNTL groups during development. (**A**) DTC5/DTC1; (**B**) WTC5/WTC1. Ingenuity Pathway Analysis (IPA) was used to identify significant effects on canonical pathways ($-\log(p\text{-value}) > 1.3$). Percentage of genes down-regulated (green), up-regulated (red), not represented in MAKER gene set (white) are shown for the 5 most significant pathways ($-\log(p$-values) in yellow) in each comparison; both significant and non-significant DE are included. Total number of genes in each pathway are in bold. Domesticated turkey (DT), wild turkey (WT), control (CNTL), control 1 day (C1), control 5 days (C5).

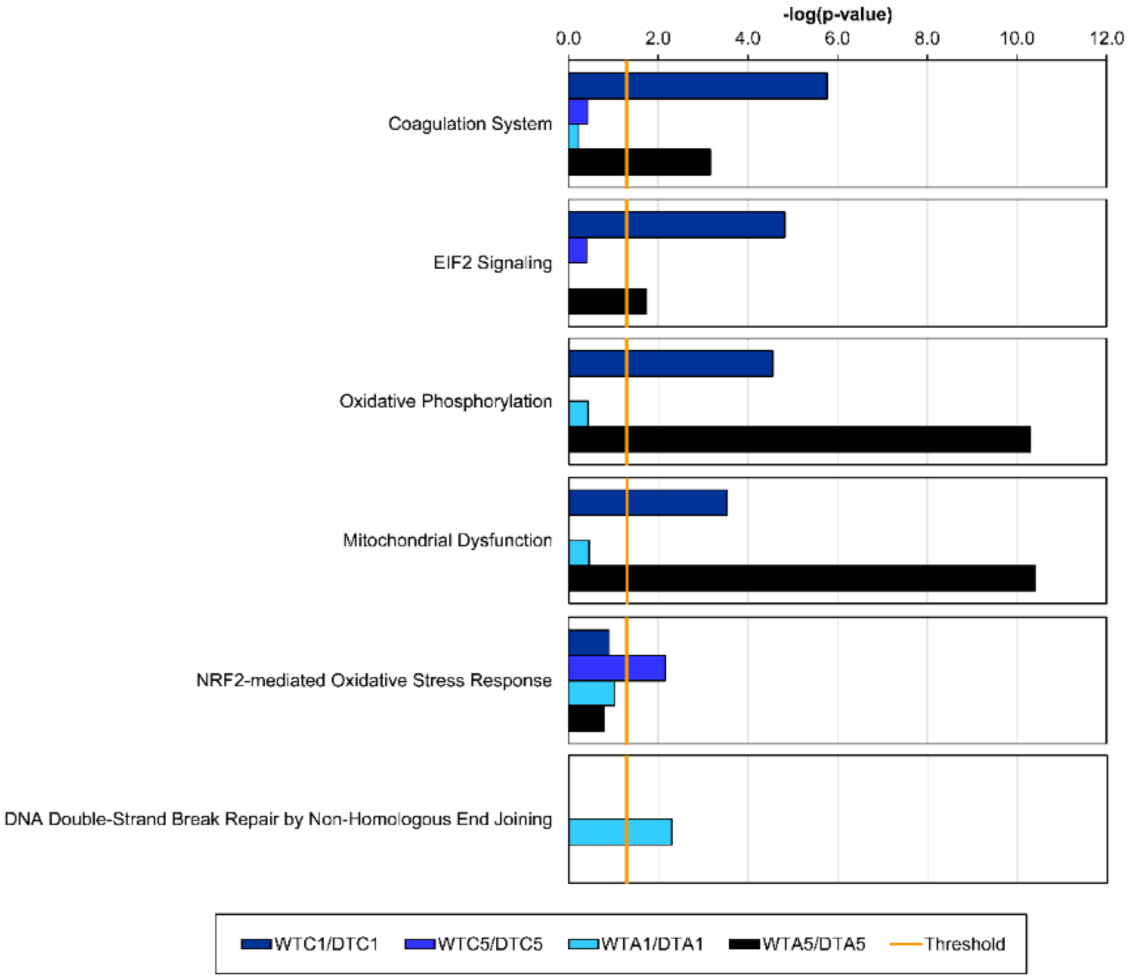

Figure S7. Significance of pathway associations vary in WT versus DT comparisons. Ingenuity Pathway Analysis (IPA) assigned *p*-values to canonical pathways based on differential expression (DE). Bar plot provides 6 example pathways with variable significance between the WTC1/DTC1 (dark blue), WTC5/DTC5 (bright blue), WTA1/DTA1 (light blue), and WTA5/DTA5 (black) comparisons. Pathway associations must have a −log(*p*-value) > 1.3 (threshold line, yellow) to be considered significant. Domesticated turkey (DT), wild turkey (WT), control 1 day (C1), control 5 days (C5), aflatoxin B_1 1 day (A1), aflatoxin B_1 5 days (A5).

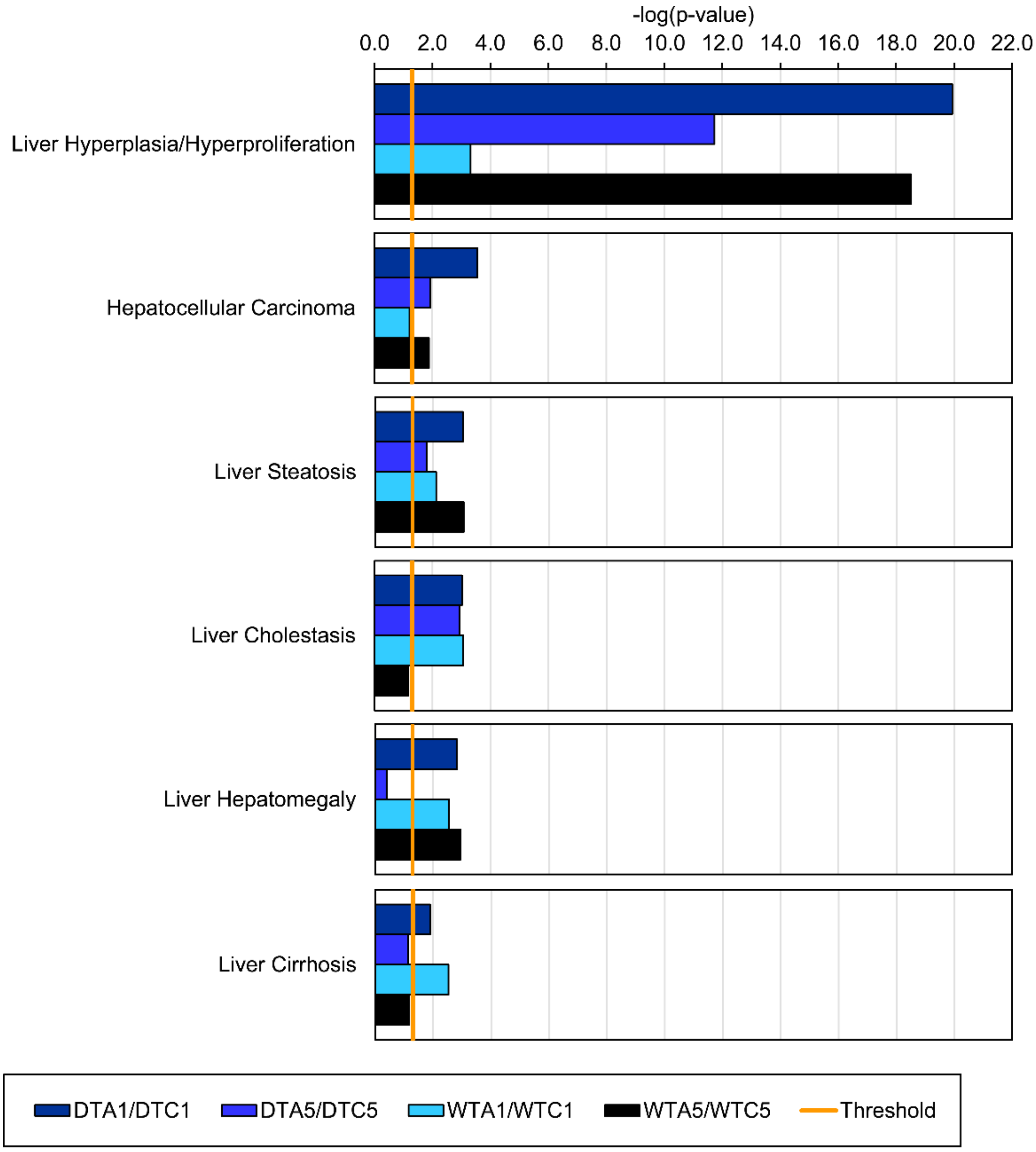

Figure S8. Significance of hepatotoxicity associations in AFB versus CNTL comparisons. Ingenuity Pathway Analysis (IPA) assigned *p*-values to toxicological functions based on differential expression (DE). Bar plot provides the 6 most significant hepatotoxic outcomes across the DTA1/DTC1 (dark blue), DTA5/DTC5 (bright blue), WTA1/WTC1 (light blue), and WTA5/WTC5 (black) comparisons. Functional associations must have a −log(*p*-value) > 1.3 (threshold line, yellow) to be considered significant. Domesticated turkey (DT), wild turkey (WT), control (CNTL), control 1 day (C1), control 5 days (C5), aflatoxin B_1 (AFB), aflatoxin B_1 1 day (A1), aflatoxin B1 5 days (A5).

Table S1. Post hoc comparisons of mean egg, embryo and liver weights.

Group	Mean	St Dev	Group	Mean	St Dev	*p*-Value
			Egg Weights			
DTA1	81.13	±8.49	DTC1	79.56	±5.62	1.00
DTA5	82.16	±5.56	DTC5	76.60	±5.71	0.68
DTC5	76.60	±5.71	DTC1	79.56	±5.62	0.98
DTA5	82.16	±5.56	DTA1	81.13	±8.49	1.00
WTA1	74.70	±4.51	WTC1	66.77	±8.75	0.69
WTA5	60.18	±3.68	WTC5	66.30	±10.52	0.84
WTC5	66.30	±10.52	WTC1	66.77	±8.75	1.00
WTA5	60.18	±3.68	WTA1	74.70	±4.51	0.081
WTA1	74.70	±4.51	DTA1	81.13	±8.49	0.78
WTA5	60.18	±3.68	DTA5	82.16	±5.56	<0.001
WTC1	66.77	±8.75	DTC1	79.56	±5.62	0.051
WTC5	66.30	±10.52	DTC5	76.60	±5.71	0.18
			Embryo Weights			
DTA1	17.67	±0.49	DTC1	16.58	±0.97	0.94
DTA5	32.60	±2.86	DTC5	30.70	±1.93	0.53
DTC5	30.70	±1.93	DTC1	16.58	±0.97	< 0.001
DTA5	32.60	±2.86	DTA1	17.67	±0.49	< 0.001
WTA1	19.08	±1.17	WTC1	18.71	±1.09	1.00
WTA5	24.83	±0.46	WTC5	29.59	±4.46	0.023
WTC5	29.59	±4.46	WTC1	18.71	±1.09	< 0.001
WTA5	24.83	±0.46	WTA1	19.08	±1.17	0.0078
WTA1	19.08	±1.17	DTA1	17.67	±0.49	0.94
WTA5	24.83	±0.46	DTA5	32.60	±2.86	< 0.001
WTC1	18.71	±1.09	DTC1	16.58	±0.97	0.58
WTC5	29.59	±4.46	DTC5	30.70	±1.93	0.97
			Liver Weights			
DTA1	0.28	±0.03	DTC1	0.26	±0.03	1.00
DTA5	0.53	±0.22	DTC5	0.65	±0.13	0.047
DTC5	0.65	±0.13	DTC1	0.26	±0.03	<0.001
DTA5	0.53	±0.22	DTA1	0.28	±0.03	<0.001
WTA1	0.33	±0.04	WTC1	0.34	±0.04	1.00
WTA5	0.46	±0.06	WTC5	0.64	±0.07	0.016
WTC5	0.64	±0.07	WTC1	0.34	±0.04	<0.001
WTA5	0.46	±0.06	WTA1	0.33	±0.04	0.20
WTA1	0.33	±0.04	DTA1	0.28	±0.03	0.92
WTA5	0.46	±0.06	DTA5	0.53	±0.22	0.68
WTC1	0.34	±0.04	DTC1	0.26	±0.03	0.54
WTC5	0.64	±0.07	DTC5	0.65	±0.13	1.00
			Relative Liver Weights			
DTA1	1.56	±0.17	DTC1	1.56	±0.12	1.00
DTA5	1.64	±0.22	DTC5	2.12	±0.37	0.0086
DTC5	2.12	±0.37	DTC1	1.56	±0.12	0.0015
DTA5	1.64	±0.22	DTA1	1.56	±0.17	1.00
WTA1	1.74	±0.26	WTC1	1.81	±0.20	1.00
WTA5	1.87	±0.25	WTC5	2.21	±0.34	0.41
WTC5	2.21	±0.34	WTC1	1.81	±0.20	0.24
WTA5	1.87	±0.25	WTA1	1.74	±0.26	0.99
WTA1	1.74	±0.26	DTA1	1.56	±0.17	0.93
WTA5	1.87	±0.25	DTA5	1.64	±0.22	0.71
WTC1	1.81	±0.20	DTC1	1.56	±0.12	0.61
WTC5	2.21	±0.34	DTC5	2.12	±0.37	1.00

Non-significant *p*-values (≥0.05) from multiple comparisons tests are shown in grey. Domesticated turkey (DT), wild turkey (WT), control 1 day (C1), control 5 days (C5), aflatoxin B_1 1 day (A1), aflatoxin B_1 5 days (A5).

Table S3. Distribution and expression of the MAKER gene set across the turkey genome (UMD 5.0).

Chromosome	Size (Mb)	Total Genes	BLAST Annotated Genes	Gene Density [1]	Genes Expressed in		
					DT	WT	Both
1	190.7	2228	2109	41.0%	2155	2160	2174
2	109.7	1395	1295	44.0%	1354	1352	1365
3	93.2	879	813	43.5%	847	841	853
4	70.7	874	813	50.8%	848	844	854
5	59.0	1024	939	47.7%	996	997	1001
6	50.7	699	630	47.9%	660	652	662
7	36.3	584	543	49.6%	565	562	569
8	34.7	569	529	48.5%	551	548	554
9	19.1	379	348	46.9%	369	369	371
10	29.2	620	570	52.3%	609	602	611
11	23.7	524	473	50.3%	502	501	505
12	19.6	447	415	54.5%	430	434	436
13	19.1	409	388	49.1%	403	401	404
14	19.5	358	330	47.4%	349	347	353
15	17.5	400	366	38.6%	385	384	390
16	14.9	462	435	51.4%	449	449	453
17	13.1	394	372	53.8%	386	385	388
18	0.3	32	31	82.7%	31	30	31
19	10.3	315	288	55.3%	299	299	300
20	10.7	325	309	48.8%	313	315	316
21	9.7	359	342	57.1%	346	346	350
22	13.7	390	362	43.3%	376	378	381
23	6.7	291	274	52.7%	276	276	281
24	4.1	119	112	58.0%	117	117	118
25	5.6	230	221	44.0%	225	224	225
26	6.4	198	173	43.0%	192	189	192
27	1.4	106	105	58.3%	102	104	104
28	5.0	261	249	58.1%	253	249	255
29	4.3	240	231	58.4%	231	230	234
30	4.6	287	277	57.9%	281	279	281
W	0.3	2	2	83.6%	2	2	2
Z	68.5	772	739	40.1%	753	743	755
Unassigned	177.0	2093	2034	3.2%	1638	1602	1672
Total Genome	1149.2	18,265	17,117	39.4%	17,293	17,211	17,440

Domesticated turkey (DT), wild turkey (WT). [1] Total length of genes (including introns and UTRs) per chromosome/chromosome size.

Table S6. Significant DE genes involved in NRF2-mediated responses in AFB versus CNTL groups.

Gene ID	Gene	DTA1/DTC1		DTA5/DTC5		WTA1/WTC1		WTA5/WTC5	
		Log_2FC	*q*-Value	Log_2FC	*q*-Value	Log_2FC	*q*-Value	Log_2FC	*q*-Value
Upstream Cytoplasmic									
T_ALT_S_009346	*ACTA2*	0.50	1.05×10^{-2}	0.05	9.24×10^{-1}	0.13	9.69×10^{-1}	0.22	5.13×10^{-1}
T_ALT_S_010243	*ACT5*	−0.03	8.66×10^{-1}	−0.01	9.62×10^{-1}	0.04	9.92×10^{-1}	0.43	5.97×10^{-3}
T_ALT_S_006365	*X10IF2AK3*	−0.37	3.08×10^{-3}	−0.06	8.11×10^{-1}	−0.22	6.14×10^{-1}	−0.04	9.06×10^{-1}
T_ALT_S_010902	*MAP2K3*	0.57	1.64×10^{-3}	0.05	9.03×10^{-1}	0.24	8.44×10^{-1}	0.34	2.37×10^{-1}
T_ALT_S_010558	*MAP2K5*	−0.27	1.15×10^{-2}	0.01	9.67×10^{-1}	−0.24	6.09×10^{-1}	−0.12	6.12×10^{-1}
T_ALT_S_011089	*MAPK1*	−0.20	1.77×10^{-2}	0.06	6.87×10^{-1}	−0.15	7.67×10^{-1}	−0.25	2.44×10^{-2}
T_ALT_S_015036	*MAPK14* [1]	−0.23	4.07×10^{-2}	−0.17	2.21×10^{-1}	−0.03	9.90×10^{-1}	−0.26	6.29×10^{-2}
T_ALT_S_015037	*MAPK14* [1]	0.94	5.73×10^{-13}	−0.07	7.93×10^{-1}	0.45	3.05×10^{-1}	0.70	3.96×10^{-4}
T_ALT_S_009331	*MAPK8*	−0.37	1.59×10^{-3}	−0.30	2.56×10^{-2}	−0.11	9.41×10^{-1}	−0.18	3.14×10^{-1}
T_ALT_S_010038	*PIK3CA*	−0.54	1.84×10^{-3}	−0.29	2.81×10^{-1}	−0.27	6.42×10^{-1}	−0.30	1.50×10^{-1}
T_ALT_S_014476	*PIK3CD*	−0.33	4.13×10^{-2}	0.20	3.18×10^{-1}	0.36	6.12×10^{-1}	0.20	5.08×10^{-1}
T_ALT_S_006672	*PIK3R1* [1]	−0.05	8.48×10^{-1}	−0.53	1.79×10^{-2}	−0.26	6.21×10^{-1}	−0.67	6.42×10^{-3}
T_ALT_S_006673	*PIK3R1* [1]	−0.09	7.56×10^{-1}	−0.67	2.14×10^{-2}	−0.06	9.89×10^{-1}	−0.31	3.35×10^{-1}
T_ALT_S_011518	*PRKCD*	0.12	5.36×10^{-1}	−0.46	4.06×10^{-2}	−0.05	9.92×10^{-1}	−0.40	1.16×10^{-1}
T_ALT_S_002624	*PRKCX10*	−0.71	8.70×10^{-3}	0.31	4.30×10^{-1}	0.15	9.63×10^{-1}	−0.44	2.47×10^{-1}
T_ALT_S_004522	*PRKCH* [1]	0.50	1.42×10^{-1}	0.41	2.16×10^{-1}	−0.28	8.22×10^{-1}	1.27	3.87×10^{-5}
T_ALT_S_004523	*PRKCH* [1]	0.27	NA	0.38	3.25×10^{-1}	0.02	NA	1.39	4.58×10^{-5}
T_ALT_S_010069	*PRKCI*	−0.27	1.01×10^{-2}	0.07	7.63×10^{-1}	−0.01	9.97×10^{-1}	−0.02	9.47×10^{-1}
T_ALT_S_004239	*PRKD1*	−0.21	2.82×10^{-1}	0.20	2.85×10^{-1}	−0.11	9.69×10^{-1}	−0.41	4.01×10^{-2}
Nuclear									
T_ALT_S_002034	*ATM*	−0.25	2.01×10^{-2}	−0.26	2.63×10^{-2}	−0.09	9.38×10^{-1}	−0.06	7.25×10^{-1}
T_ALT_S_001339	*BACH1*	−0.51	3.24×10^{-3}	−0.36	3.18×10^{-2}	−0.30	4.15×10^{-1}	−0.63	1.12×10^{-4}
T_ALT_S_014852	*CDC34*	−0.33	9.37×10^{-2}	−0.64	8.97×10^{-4}	0.05	9.93×10^{-1}	0.04	8.76×10^{-1}
T_ALT_S_010676	*CRX10BBP*	0.45	2.35×10^{-5}	0.07	6.94×10^{-1}	−0.18	9.21×10^{-1}	0.67	9.17×10^{-3}
T_ALT_S_009069	*DNAJB4*	−0.24	4.29×10^{-2}	−0.20	1.46×10^{-1}	−0.06	9.89×10^{-1}	−0.08	7.75×10^{-1}
T_ALT_S_007316	*DNAJB6*	−0.35	3.03×10^{-2}	−0.25	1.06×10^{-1}	−0.12	9.49×10^{-1}	−0.22	4.65×10^{-1}
T_ALT_S_009102	*DNAJC9*	−0.35	2.39×10^{-2}	0.01	9.81×10^{-1}	−0.27	6.24×10^{-1}	−0.47	5.08×10^{-2}
T_ALT_S_000577	*X10P300*	−0.04	8.35×10^{-1}	−0.14	4.30×10^{-1}	0.13	8.84×10^{-1}	−0.36	3.48×10^{-2}
T_ALT_S_001124	*GSK3B*	0.15	2.86×10^{-1}	0.23	2.25×10^{-1}	0.04	9.93×10^{-1}	0.33	4.84×10^{-2}
T_ALT_S_008102	*NFX102L2*	0.58	1.84×10^{-7}	0.33	3.70×10^{-2}	0.52	1.09×10^{-3}	0.21	1.44×10^{-1}
T_ALT_S_002690	*PRKD3*	−0.04	8.59×10^{-1}	0.06	8.68×10^{-1}	0.06	9.84×10^{-1}	−0.41	4.26×10^{-2}
T_ALT_S_008084	*UBX102X 103*	−0.23	1.83×10^{-1}	−0.10	6.92×10^{-1}	0.03	9.93×10^{-1}	−0.59	1.34×10^{-2}

Table S6. *Cont.*

				Downstream Cytoplasmic					
T_ALT_S_008999	*AKR1A1*	0.34	3.18×10^{-2}	0.07	7.76×10^{-1}	0.25	8.07×10^{-1}	0.69	5.58×10^{-5}
T_ALT_S_014672	*AKR7A2*	0.47	1.15×10^{-2}	0.14	4.91×10^{-1}	0.34	5.55×10^{-1}	0.18	3.37×10^{-1}
T_ALT_S_008024	*AOX1*	0.28	3.65×10^{-2}	0.00	9.96×10^{-1}	−0.02	9.95×10^{-1}	−0.04	8.86×10^{-1}
T_ALT_S_003995	*CAT*	−0.18	3.15×10^{-1}	−0.44	3.49×10^{-4}	−0.18	8.54×10^{-1}	−0.30	1.58×10^{-1}
T_ALT_S_010665	*DNAJA3*	0.40	1.15×10^{-3}	−0.03	8.79×10^{-1}	0.07	9.78×10^{-1}	0.21	1.66×10^{-1}
T_ALT_S_009842	*DNAJB11*	−0.32	7.44×10^{-3}	−0.08	NA	−0.39	2.36×10^{-1}	0.31	1.15×10^{-1}
T_ALT_S_008078	*DNAJC10*	−0.39	1.01×10^{-3}	−0.02	9.65×10^{-1}	−0.05	9.91×10^{-1}	0.18	4.09×10^{-1}
T_ALT_S_014426	*DNAJC11*	0.26	4.81×10^{-2}	−0.06	7.63×10^{-1}	−0.20	8.47×10^{-1}	0.20	3.88×10^{-1}
T_ALT_S_007708	*DNAJC13*	0.03	8.56×10^{-1}	−0.40	6.56×10^{-3}	0.07	9.80×10^{-1}	0.08	7.48×10^{-1}
T_ALT_S_014061	*DNAJC18*	−0.02	8.95×10^{-1}	−0.02	9.44×10^{-1}	−0.24	5.93×10^{-1}	−0.42	1.81×10^{-2}
T_ALT_S_002734	*X10PHX1*	0.63	5.74×10^{-4}	−0.06	8.95×10^{-1}	0.03	9.93×10^{-1}	0.57	2.36×10^{-3}
T_ALT_S_003948	*FTH1*	0.10	6.77×10^{-1}	0.38	4.52×10^{-2}	−0.10	9.41×10^{-1}	0.12	7.29×10^{-1}
T_ALT_S_003347	*GSTA3* [1]	1.60	1.47×10^{-10}	0.74	1.79×10^{-2}	0.48	3.63×10^{-1}	0.52	2.34×10^{-1}
T_ALT_S_003348	*GSTA3* [1]	1.52	2.87×10^{-9}	0.85	4.31×10^{-3}	0.39	5.52×10^{-1}	0.23	6.61×10^{-1}
T_ALT_S_003346	*GSTA3* [1]	1.11	9.74×10^{-5}	0.40	2.66×10^{-1}	0.36	5.83×10^{-1}	0.80	4.76×10^{-2}
T_ALT_S_003345	*GSTA4*	0.82	8.65×10^{-7}	0.47	2.82×10^{-2}	0.35	6.56×10^{-1}	0.42	4.09×10^{-2}
T_ALT_S_001023	*GSTK1*	0.07	6.99×10^{-1}	−0.31	2.39×10^{-2}	−0.26	5.83×10^{-1}	−0.11	6.34×10^{-1}
T_ALT_S_009475	*GSTO1*	0.13	5.87×10^{-1}	0.06	8.65×10^{-1}	−0.28	3.27×10^{-1}	1.07	4.63×10^{-3}
T_ALT_S_011992	*HX10RPUD1*	−0.25	3.76×10^{-1}	0.69	3.75×10^{-4}	−0.20	9.21×10^{-1}	−0.26	3.11×10^{-1}
T_ALT_S_000662	*HMOX1*	0.29	1.91×10^{-1}	−0.14	7.31×10^{-1}	0.32	3.84×10^{-1}	0.85	1.48×10^{-9}
T_ALT_S_005601	*MGST2*	0.84	6.76×10^{-7}	0.14	6.23×10^{-1}	0.41	2.18×10^{-1}	0.37	8.19×10^{-2}
T_ALT_S_008700	*MGST3*	0.56	9.79×10^{-5}	0.10	7.83×10^{-1}	0.33	2.55×10^{-1}	0.73	4.64×10^{-4}
T_ALT_S_012166	*NQO1*	0.63	2.64×10^{-6}	0.03	8.87×10^{-1}	0.57	1.81×10^{-3}	0.02	9.39×10^{-1}
T_ALT_S_004785	*NQO2*	0.24	2.42×10^{-1}	0.63	1.94×10^{-4}	0.00	1.00	0.73	6.78×10^{-6}
T_ALT_S_009000	*PRDX1*	0.49	2.13×10^{-3}	0.10	6.39×10^{-1}	0.10	9.69×10^{-1}	0.66	1.03×10^{-4}
T_ALT_S_016734	*STIP1*	−0.03	8.93×10^{-1}	−0.19	2.64×10^{-1}	−0.14	8.98×10^{-1}	0.36	9.48×10^{-3}
T_ALT_S_007072	*TXN*	0.09	5.73×10^{-1}	−0.62	2.26×10^{-3}	−0.05	9.80×10^{-1}	−0.07	6.80×10^{-1}
T_ALT_S_000674	*TXNRD1* [1]	0.61	1.32×10^{-2}	0.15	7.63×10^{-1}	0.54	2.55×10^{-1}	0.45	2.82×10^{-1}
T_ALT_S_011677	*TXNRD1* [1]	−0.30	2.07×10^{-3}	0.00	9.99×10^{-1}	0.00	9.99×10^{-1}	−0.16	3.06×10^{-1}
T_ALT_S_007907	*UGT1A1*	0.62	2.35×10^{-5}	0.06	8.19×10^{-1}	0.15	9.05×10^{-1}	0.34	8.16×10^{-3}
				Extracellular/Plasma Membrane					
T_ALT_S_009396	*MRP2*	0.16	3.07×10^{-1}	−0.32	3.68×10^{-2}	0.24	4.82×10^{-1}	0.24	2.59×10^{-1}
T_ALT_S_006174	*SOD3*	0.33	3.12×10^{-1}	0.17	7.08×10^{-1}	0.07	9.92×10^{-1}	0.58	2.25×10^{-2}

Genes were identified using Ingenuity Pathway Analysis (IPA). Log_2 fold change (Log_2FC) and FD-adjusted *p*-values (*q*-values) were determined in DESeq2 [30]. Differential expression (DE), domesticated turkey (DT), wild turkey (WT), control (CNTL), control 1 day (C1), control 5 days (C5), aflatoxin B_1 (AFB), aflatoxin B_1 1 day (A1), aflatoxin B_1 5 days (A5), no statistics due to low read counts (NA). [1] Multiple genes in the MAKER gene set had significant DE and annotated to the same reference.
